# Predictors of shorter- and longer-term mortality after COVID-19 presentation among dialysis patients: parallel use of machine learning models in Latin and North American countries

**DOI:** 10.1186/s12882-022-02961-x

**Published:** 2022-10-22

**Authors:** Adrián M. Guinsburg, Yue Jiao, María Inés Díaz Bessone, Caitlin K. Monaghan, Beatriz Magalhães, Michael A. Kraus, Peter Kotanko, Jeffrey L. Hymes, Robert J. Kossmann, Juan Carlos Berbessi, Franklin W. Maddux, Len A. Usvyat, John W. Larkin

**Affiliations:** 1 Fresenius Medical Care Latin America, Rio de Janeiro, Brazil; 2 Fresenius Medical Care, Global Medical Office, 920 Winter Street, Waltham, MA 02451 USA; 3 Fresenius Medical Care North America, Waltham, USA; 4 Renal Research Institute, New York, USA; 5 Icahn School of Medicine at Mount Sinai, New York, USA; 6 Fresenius Medical Care AG & Co. KGaA, Global Medical Office, Bad Homburg, Germany

**Keywords:** COVID-19, Hemodialysis, Mortality Risk, Machine Learning, Prediction Model, Multinational

## Abstract

**Background:**

We developed machine learning models to understand the predictors of shorter-, intermediate-, and longer-term mortality among hemodialysis (HD) patients affected by COVID-19 in four countries in the Americas.

**Methods:**

We used data from adult HD patients treated at regional institutions of a global provider in Latin America (LatAm) and North America who contracted COVID-19 in 2020 before SARS-CoV-2 vaccines were available. Using 93 commonly captured variables, we developed machine learning models that predicted the likelihood of death overall, as well as during 0–14, 15–30, > 30 days after COVID-19 presentation and identified the importance of predictors. XGBoost models were built in parallel using the same programming with a 60%:20%:20% random split for training, validation, & testing data for the datasets from LatAm (Argentina, Columbia, Ecuador) and North America (United States) countries.

**Results:**

Among HD patients with COVID-19, 28.8% (1,001/3,473) died in LatAm and 20.5% (4,426/21,624) died in North America. Mortality occurred earlier in LatAm versus North America; 15.0% and 7.3% of patients died within 0–14 days, 7.9% and 4.6% of patients died within 15–30 days, and 5.9% and 8.6% of patients died > 30 days after COVID-19 presentation, respectively. Area under curve ranged from 0.73 to 0.83 across prediction models in both regions. Top predictors of death after COVID-19 consistently included older age, longer vintage, markers of poor nutrition and more inflammation in both regions at all timepoints. Unique patient attributes (higher BMI, male sex) were top predictors of mortality during 0–14 and 15–30 days after COVID-19, yet not mortality > 30 days after presentation.

**Conclusions:**

Findings showed distinct profiles of mortality in COVID-19 in LatAm and North America throughout 2020. Mortality rate was higher within 0–14 and 15–30 days after COVID-19 in LatAm, while mortality rate was higher in North America > 30 days after presentation. Nonetheless, a remarkable proportion of HD patients died > 30 days after COVID-19 presentation in both regions. We were able to develop a series of suitable prognostic prediction models and establish the top predictors of death in COVID-19 during shorter-, intermediate-, and longer-term follow up periods.

**Supplementary Information:**

The online version contains supplementary material available at 10.1186/s12882-022-02961-x.

## Background

People with kidney failure treated by dialysis are at a high risk of experiencing serious complications if affected by COVID-19. Reports have estimated 40% to 70% of dialysis patients who contracted COVID-19 were hospitalized and 11% to 34% died during timeframes before SARS-CoV-2 vaccines were available [1–9]. During 2020, the mortality rate in the United States dialysis population was estimated to have increased by 18% compared to 2019 [10]. The majority assessments of mortality in dialysis patients with COVID-19 have limited follow up timeframes for assessing outcomes. Although useful, the timeframes generally investigated do not provide an understating of outcomes overall, as well as in distinct shorter and longer periods after COVID-19. Ultimately, this might be limiting our understanding of the profiles and predictors of outcomes in this special population.

In many countries, SARS-CoV-2 vaccines have fortunately become readily available and are being rolled out to the communities [11, 12]. Nonetheless, SARS-CoV-2 vaccines have been shown to create a smaller antibody response among dialysis patients [13, 14], and a proportion of the population has not been vaccinated for SARS-CoV-2, and may never be due to various reasons (e.g. medical/religious contraindications, vaccine hesitancy) [11, 12, 15, 16]. Further establishment of models to identify the predictors of outcomes in unvaccinated dialysis patients continues to be warranted, and as sufficient follow up data becomes available, investigations determining the profiles and predictors of mortality in vaccinated dialysis patients will also be needed.

Through experiences in direct patient care in the pandemic, the physician authors made anecdotal observations that dialysis patients with COVID-19 generally experienced the outcome of death either very quickly (within 14 days), or after prolonged periods of intensive care (often > 30 days). Ultimately, it was hypothesized this might be signaling distinct causes and predictors of early or prolonged mortality in COVID-19. This investigation aimed to evaluate the profiles and predictors of mortality in hemodialysis (HD) patients with COVID-19, overall, as well as considering shorter-, intermediate-, and longer-term follow up periods.

## Methods

### Patient cohorts

We used real-world data from HD patients treated at regional institutions of a global provider in Latin America (LatAm; Fresenius Medical Care Latin America, Rio de Janeiro, Brazil) and North America (Fresenius Medical Care North America, Waltham, United States) from 01-July-2019 to 31-December-2020 to conduct side-by-side analyses of the profiles and predictors of mortality overall, as well as within 0–14, 15–30, > 30 days after COVID-19 presentation.

In LatAm and North America, we used data collected during the provision of routine medical care in dialysis patients. All data was de-identified for the purposes of the parallel analyses. The EuCliD database was used for capturing data in the Latin America cohort as part of Fresenius Medical Care's quality improvement and management programs in all NephroCare clinics utilizing EuCLiD [17]. EuCLiD governance has established protocols and procedures for use of clinical data from NephroCare clinics for secondary research purposes. Data was only collected from patients who provided informed consent for their data to be collected into EuCliD and the data was de-identified by the LatAm investigator. The Fresenius Medical Care North America Knowledge Center Data Warehouse was used for capturing data in the North America cohort from clinics in the Fresenius Kidney Care network. In North America, data was collected from patients treated in the United States under a protocol approved by New England Independent Review Board (NEIRB; Needham Heights, MA, United States); NEIRB determined the analysis of the North America cohort was exempt due to use of data de-identified by the North America investigator that no longer contained protected health information and consent was not required per title 45 of the United States Code of Federal Regulations part 46.104(d)(4) (NEIRB# 1–1439054-1). The analysis in each region was conducted in accordance with the Declaration of Helsinki.

### Patient eligibility

We included data from adult (age ≥ 18 years) patients with kidney failure who were suspected to have COVID-19 before 02-Dec-2020 and received ≥ 1 outpatient HD treatment (inclusive of hemodiafiltration) before COVID-19 presentation and did not change to a home dialysis modality during the observation period. We excluded data from patients under investigation who were found to have a negative SARS-CoV-2 test result, or patients who were in close contact to someone with known COVID-19, never presented with symptoms, and were not tested. We also excluded data from patients who received outpatient HD for acute kidney failure, as well as patients who were known to be pregnant during the observation period.

### Dependent variables

The primary outcome (dependent variable) was all-cause death any time after COVID-19 presentation. The time at risk started on the first date of COVID-19 suspicion where patients presented with signs and symptoms. We defined a 30-day minimum follow up period for evaluation of outcomes across the observation period (i.e. COVID-19 suspicion date before 02 Dec 2020).

Further sub-analyses of the primary outcome considered all-cause death within 0–14, 15–30, > 30 days after COVID-19 presentation. We used the same logic for time at risk and minimum follow up as with death any time after COVID-19 presentation. Patients who had a death event in a preceding period were censored from the dataset for analysis performed in the subsequent predefined follow-up period. Therefore, patients who died within 0–14 days after COVID-19 presentation were removed from analyses of outcomes 15–30 and > 30 days after COVID-19 presentation. Consistently, patients who died during 15–30 days after COVID-19 presentation were removed from analyses of outcomes > 30 days after COVID-19 presentation.

### Independent variables

We used various patient characteristics, clinical parameters, and laboratories (independent variables; *n* = 93) to define the characteristics of the cohorts and investigate the predictors of death after COVID-19 presentation. Patient characteristics included age, sex, body mass index (BMI), dialysis vintage, etiology of kidney failure (diabetic nephropathy, hypertensive nephrosclerosis, or other), comorbidities (diabetes, hypertension, heart failure, ischemic heart disease, liver disease, cancer, and chronic obstructive pulmonary disease (COPD)), and country as of the first date of suspicion/presentation with COVID-19, as well as continuous dialysis catheter exposure ≤ 90, ≤ 120, and ≤ 180 days before COVID-19 presentation.

Clinical parameters included pre-/post-HD systolic blood pressure (SBP), diastolic blood pressure (DBP), pulse, body temperature, and weight, as well as the prescribed dry weight and interdialytic weight gain (IDWG). Laboratories included pre-HD albumin, calcium, corrected calcium, creatinine, phosphate, intact parathyroid hormone (iPTH), hemoglobin, ferritin, transferrin saturation (TSAT), white blood cell (WBC) count, and WBC differential (% platelets, % lymphocytes, % neutrophils). All independent variables considered were captured and available for patients treated in the Latin and North America countries included in the parallel analyses. The clinical parameters (e.g., vital signs and weight measures) were universally collected before and after HD for all patients in both regional cohorts. There were some differences in the frequencies of select laboratories with some being measured less frequently in Latin versus North America countries. For instance, pre-HD albumin was measured on a quarterly basis in Latin America and a monthly basis in North America.

**Fig. 1:**
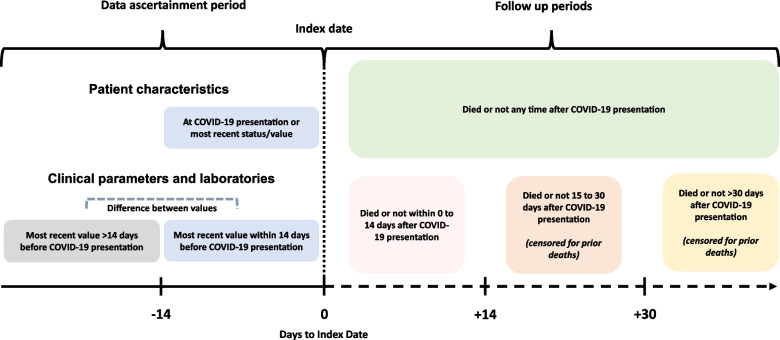
Timeframe of data ascertainment and follow up of outcomes after COVID-19 presentation (index date)

All clinical parameters and laboratory values considered the most recent value within 14 days before COVID-19 presentation, the most recent value > 14 days prior to COVID-19 presentation, and the change between the values within 14 days and > 14 days prior to COVID-19 presentation (Fig. 1). These timepoints were selected based off expert knowledge in the domain of medicine and physiology and a prior investigation that estimated the timing of physiological disturbances during the onset of COVID-19 [18].

A past work identified disturbances in physiology start about 14 days before COVID-19, with the most meaningful changes in clinical and laboratory values being seen at presentation with the first signs and symptoms of the disease [18]. Therefore, the most recent value within 14 days of COVID-19 was chosen to provide a representation of the patient’s clinical status at presentation with signs of symptoms that led to identification of COVID-19. This prior analysis also showed that clinical and laboratory values > 14 days before COVID-19 presentation were representative of each patient’s “normal” physiology before the onset of COVID-19 [18]. Ultimately, this design for predictor variable timing was chosen to show the extent that disturbances in clinical and laboratory values during COVID-19 onset associate with a death event, as well as show the extent that the historic clinical status associates with a death event.

### Statistical analysis

#### Descriptive statistics

The patient characteristics in the LatAm and North America cohorts were tabulated by region, as well as stratified by the groups who died or survived after COVID-19. We reported the count and proportion of categorical variables and mean and standard deviation (SD) of continuous variables.

#### Machine learning model development

Given the knowledge on risk factors for mortality in the dialysis population is sparse, has not included continuous data on laboratories and HD treatments, and has not assessed temporal changes in predictors before and longer follow up times after COVID-19 presentation, we decided to use an advanced data driven approach to establish the predictors of mortality after COVID-19. This included developing a series of machine learning prediction models using Python software (Python Software Foundation, Delaware, United States) with the XGBoost package [19] to predict the likelihood of death after COVID-19 presentation and identify the importance of predictor variables.

For parallel model development in LatAm and North America, we used a 60%:20%:20% random split of the data on patients who died anytime during follow-up (i.e. positive outcome group) for the training, validation, and testing datasets respectively. Data from survivors throughout follow-up (i.e. negative outcome group) were randomly split between the datasets. Down sampling methods in the negative outcome cases were investigated to optimize the models’ ability to learn to identify the outcome from predictor variables considering a 1:1 through 1:6 ratio in the training and validation datasets. Based on our assessments, we chose to down sample the negative outcome cases in only the training dataset to provide a 1:2 ratio of positive to negative outcome cases (i.e. for each patient who died we randomly included 2 patients who survived in the training dataset). The validation and testing datasets were not down sampled and represented the incidence of COVID-19 death observed in the overall HD population in each world region.

The same methods for data splits and sampling were performed for the sub-analysis models developed to predict the likelihood of death in 0–14, 15–30, > 30 days after COVID-19 presentation. In these sub-analysis models, we removed patients who died within 14 days after COVID-19 from the positive outcome group for creating the datasets for the models developed to determine the risk of death during 15–30 or > 30 days after COVID-19 presentation. Furthermore, we removed patients who died within 30 days after COVID-19 from the positive outcome group for creating the datasets for the models developed to determine the probability of death > 30 days after COVID-19 presentation. The negative outcome groups consisted of data from survivors of the predefined follow-up period, and they were randomly split between the training, validation, and testing datasets for each model. All models were developed in a side-by-side manner and used same programming for datasets in LatAm and North America.

For an overview of the XGBoost logic, this non-linear machine learning model used the input (independent) variables in the training dataset to construct an array of decision trees in every possible combination to establish a series of thresholds that split variables to maximize the information gain. Decision trees were constructed iteratively by the model, and new decision trees were added to predict prior errors. The decision trees were inherently able to handle and account for missing values and imputation of null data points was not required. The model determined the presence or missingness of each variable when establishing variable splits in the decision trees for each patient. Therefore, the influence of a missing value was used for information gain in the predictions made for each patient. After the ensemble of decision trees was created using the training datasets, it was assessed using the validation datasets and hyperparameter tuning was evaluated for the overall models using a grid search and 5-fold cross validation method. After no more improvements were achieved in performance, the final ensemble of decision trees produced in the models were used for performance assessments using unseen data in the testing dataset. Hyperparameter tuning and the selection of the final hyperparameter settings in each region was based on the models that predicted mortality any time after COVID-19, and these settings were universally applied to the sub-analysis models that predicted mortality within 0–14 days, 15–30 days, and > 30 days after COVID-19. The details on the initial and final hyperparameters and tuning ranges considered are shown in Additional File 1; Supplementary Table 1.

#### Assessment of model performance

The performance of the prediction models was measured by the area under the receiver operating characteristic curve (AUC) and balanced accuracy in the training, validation, and testing datasets. The area under the precision-recall curve (AUPRC) was further evaluated in the testing dataset.

The AUC measures the rate of true and false positives classified across probability thresholds (Table 1). AUC scores are represented on a scale of 0 (lowest) to 1 (highest) with chance being a value of 0.5.

**Table 1:** Definition of true/false positive and negative predictions classified by the model in the assessment of performance

**True positives**	Patients classified as having a death event after COVID-19 by the model who are in the group with a death event after COVID-19
**False positives**	Patients classified as having a death event after COVID-19 by the model who are in the group that survived after COVID-19
**True negatives**	Patients classified as a survivor after COVID-19 by the model who are in the group that survived after COVID-19
**False negatives**	Patients classified as a survivor after COVID-19 by the model who are in the group with a death event after COVID-19

Balanced accuracy is a measure of the accuracy of the prediction that is represented as a percent and considers both the sensitivity and specificity at cutoff threshold of 0.50. This metric can reasonably estimate model performance in data with imbalanced positive and negative outcomes, and is calculated as follows:

$$Balanced\mathit\;accuracy=\;\left(\frac{\left(Sensitivity\;+\;Specificity\right)}2\right)\ast100$$

The AUPRC measures the ratio of precision for corresponding sensitivity values across probability thresholds [20]. AUPRC scores are represented on a scale of 0 (lowest) to 1 (highest) with chance equaling the fraction of positive cases in each regional group for each model (i.e., the number of patients who died in each group divided by the total number of patients in each group).

The definitions for sensitivity, specificity, and precision are provided below since these metrics are used in the calculation of balanced accuracy and the AUPRC.

Sensitivity (also known as recall) shows the rate of true positives classified by the model at a specified threshold, and the equation for this metric is as follows:

$$\begin{array}{c}Sensitivity\;=\left(\frac{true\mathit\;positives}{(true\mathit\;positives\;+\;false\mathit\;negatives)}\right)\;\ast\;100\end{array}$$

Specificity shows the rate of true negatives classified by the model at a specified threshold, and the equation for this metric is as follows:

$$Specificity=\left(\frac{true\mathit\;negatives}{(true\mathit\;negatives\;+\;false\mathit\;positives)}\right)\;\ast\;100$$

Precision shows the positive predictive value for the model at a specified threshold, and the equation for this metric is as follows:

$$Precision=\;\left(\frac{true\mathit\;positives}{\left(true\mathit\;positives\;+\mathit\;false\mathit\;positives\right)}\right)\;\ast\;100$$

The final model performance is represented by the AUC, balanced accuracy, and AUPRC for the testing dataset.

#### Assessment of the importance of predictors

We assessed the importance of individual predictor variables using Shapley (SHAP) values [21, 22] that were calculated using the SHAP Python package [23, 24]. The SHAP value determined the feature importance for each input variable by calculating the predictors influence on prediction of the outcome considering the influence of the overall combination of variables in the model.

For an overview of the logic, SHAP values were calculated for each predictor variable at each observation, representing the positive or negative impact of the observed value on the prediction of the outcome for each individual patient. The SHAP methods included and withheld the individual variables in all possible combinations. To attribute feature importance, the SHAP method calculated the mean value of all possible combinations considering differences between included and withheld variables. Notably, SHAP values show additive explanations of feature importance and are reported in log odds (i.e. the logarithm of the odds ratio). To calculate the prediction for each individual patient, the model summed the SHAP values for each variable and converted it from log odds to the probability for the occurrence of the outcome. Therefore, larger positive SHAP values increase the probability for the predicted outcome for a given patient, and larger negative SHAP values decrease the probability. The overall feature importance for each predictor variable was determined by calculating the mean absolute SHAP value across all the individual patients’ observations.

## Results

### Patient characteristics and profiles of mortality after COVID-19

**Fig. 2:**
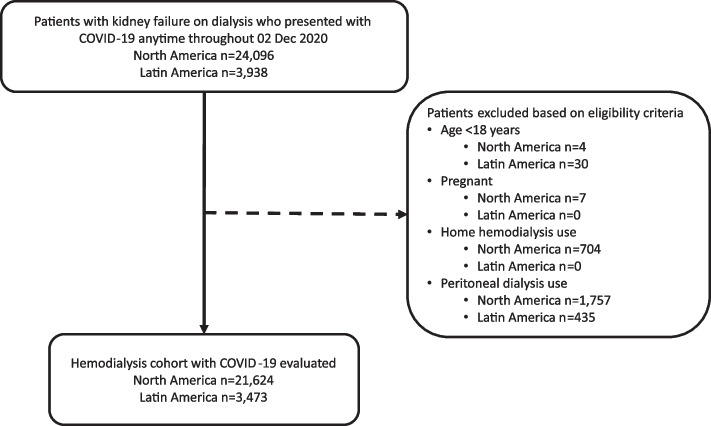
Flow diagram

We identified a cohort of 3,473 HD patients who presented with COVID-19 any time before 02 Dec 2020 in three LatAm countries (Argentina, Colombia, Ecuador), as well as a cohort of 21,624 HD patients who presented with COVID-19 during the same time in North America from the United States (Fig. 2). The demographics of patients with COVID-19 by survival status are shown in Table 2 for the LatAm and North America cohorts. On average, patients in LatAm countries had trends for being a few years younger, more often male, had a lower BMI, longer dialysis vintage, with a lower prevalence of diabetes, hypertension, and heart failure.

**Table 2:** Characteristics of HD patients who died or survived any time after COVID-19 presentation

	**Latin America**		**North America**	
	**Mean ± SD or N (%)**		**Mean ± SD or N (%)**	
**Parameter**	**Died anytime**	**Survived**	**Died anytime**	**Survived**
**Demographics**				
Age (years)	**66 ± 11.5**	57.5 ± 15.3	**68.94 ± 12.44**	61.96 ± 13.91
Male	644 (63.6)	1495 (60.1)	**2510 (56.7)**	9343 (54.3)
BMI (Kg/m^2)	26.5 ± 5.9	26.2 ± 5.9	**29.1 ± 8.8**	30.1 ± 8.8
Vintage (years)	5.23 ± 4.9	5.18 ± 4.8	**4.54 ± 4.07**	3.93 ± 4.07
**Catheter exposure**				
> 90 days	**264 (26.3)**	430 (17.5)	**999 (22.6)**	3592 (20.9)
> 120 days	**208 (20.7)**	326 (13.3)	**983 (22.2)**	3538 (20.6)
> 180 days	**199 (19.8)**	301 (12.2)	**949 (21.4)**	3452 (20.1)
**Cause of kidney failure**				
Diabetic nephropathy	**263 (26.4)**	481 (19.6)	2432 (54.9)	9295 (54.0)
Hypertensive nephrosclerosis	114 (11.4)	270 (11)	**1076 (24.3)**	4835 (28.1)
Other	**619 (62.1)**	1705 (69.4)	**915 (20.7)**	3043 (17.7)
**Comorbidities**				
Diabetes	**366 (37.9)**	645 (27.2)	**3454 (78.0)**	12,471 (72.5)
Hypertension	**376 (39)**	1032 (43.5)	**3074 (69.5)**	11,518 (67.0)
Heart failure	8 (0.8)	11 (0.4)	**1093 (24.7)**	3201 (18.6)
Ischemic heart disease	5 (0.5)	24 (1.0)	**1098 (24.8)**	3174 (18.5)
Cancer	9 (0.9)	22 (0.9)	**250 (5.6)**	610 (3.5)
COPD	4 (0.4)	14 (0.6)	**515 (11.6)**	1504 (8.7)
Liver disease	4 (0.4)	17 (0.7)	**530 (12.0)**	1770 (10.3)
**Country**				
Argentina	382	1021		
Colombia	340	822		
Ecuador	279	629		
United States			4426	17,198

*P*-values for univariate comparison of died versus survived (reference) are shown with bold font representing *p* < 0.05. All patient characteristics presented as of the COVID-19 presentation date

**Fig. 3:**
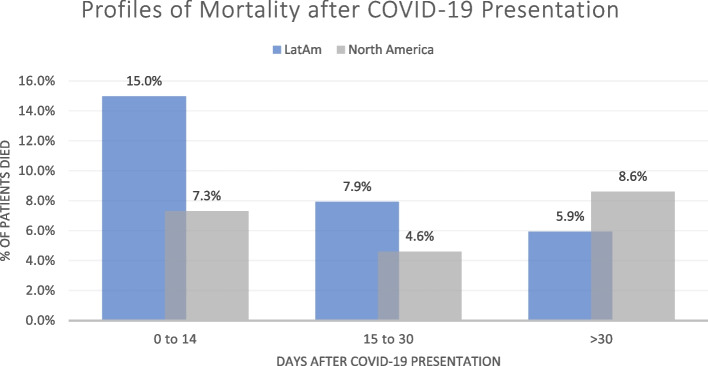
Profiles of mortality 0 to 14, 15 to 30, and > 30 days after COVID-19 presentation

In the LatAm cohort, 28.8% (1,001/3,473) patients died any time after COVID-19 during the observation period. A lower proportion of 20.5% (4,426/21,624) patients died any time after COVID-19 in the North America cohort (Table 2). There were regional differences in the timing of mortality after COVID-19, with shorter-term outcomes being more frequent in LatAm and vice versa in North America. Among HD patients with COVID-19 in LatAm and North America, 15.0% and 7.3% died within 0–14 days, 7.9% and 4.6% died within 15–30 days, and 5.9% and 8.6% died > 30 days after presentation, respectively (Fig. 3). Univariate analyses showed most demographic (Tables 2 & 3) and clinical (Tables 4, 5, 6, & 7) parameters were related to mortality in COVID-19, especially in the North America cohort.

**Table 3:** Characteristics of HD patients who died or survived 0 to 14, 15 to 30, and > 30 days after COVID-19 presentation

	**Latin America**				**North America**			
	**Mean ± SD or N (%)**				**Mean ± SD or N (%)**			
**Parameter**	**Died 0 to 14 days**	**Died 15 to 30 days**	**Died > 30 days**	**Survived**	**Died 0 to 14 days**	**Died 15 to 30 days**	**Died > 30 days**	**Survived**
**Demographics**								
Age (years)	**66.5 ± 11.2**	**65.4 ± 11.9**	**65.3 ± 11.8**	57.5 ± 15.3	**70.02 ± 12.35**	**68.77 ± 12.43**	**68.10 ± 12.47**	61.96 ± 13.91
Male	345 (64.7)	**185 (66.8)**	114 (56.3)	1495 (60.1)	**967 (61.2)**	**581 (58.8)**	**962 (51.8)**	9343 (54.3)
BMI (Kg/m^2)	**27 ± 6.3**	26.5 ± 5.5	**25.1 ± 5**	26.2 ± 5.9	**29.4 ± 8.3**	29.9 ± 11.1	**28.4 ± 7.9**	30.1 ± 8.8
Vintage (years)	5.4 ± 4.9	5.1 ± 5.3	5.0 ± 4.7	5.2 ± 4.8	**4.7 ± 4.1**	**4.7 ± 3.9**	**4.3 ± 4.1**	3.9 ± 4.1
**Catheter exposure**								
> 90 days	**124 (23.6)**	**70 (25.4)**	**70 (35.2)**	430 (17.5)	324 (20.5)	199 (20.1)	**476 (25.6)**	3592 (20.9)
> 120 days	**98 (18.6)**	**54 (19.6)**	**56 (27.1)**	326 (13.3)	312 (19.7)	199 (20.1)	**472 (25.4)**	3538 (20.6)
> 180 days	**94 (17.9)**	**51 (18.5)**	**54 (26.1)**	301 (12.2)	304 (19.2)	195 (19.7)	**450 (24.2)**	3452 (20.1)
**Cause of kidney failure**								
Diabetic nephropathy	**146 (28.1)**	62 (22.5)	**55 (27.2)**	481 (19.6)	825 (52.2)	554 (56.1)	**1053 (56.7)**	9295 (54.0)
Hypertensive nephrosclerosis	60 (11.5)	28 (10.2)	26 (12.6)	270 (11)	**376 (23.8)**	**227 (23.0)**	**473 (25.5)**	4835 (28.1)
Other	**314 (60.4)**	186 (67.3)	**119 (60.2)**	1705 (69.4)	**379 (24.0)**	**206 (20.9)**	330 (17.8)	3043 (17.7)
**Comorbidities**								
Diabetes	**199 (39.7)**	**99 (37.1)**	**68 (34.5)**	645 (27.2)	**1242 (78.6)**	**762 (77.1)**	**1450 (78.1)**	12,471 (72.5)
Hypertension	**184 (36.7)**	109 (40.8)	83 (42.5)	1032 (43.5)	**1101 (69.6)**	685 (69.3)	**1288 (69.4)**	11,518 (67.0)
Heart failure	4 (0.8)	3 (1.1)	1 (0.5)	11 (0.4)	**423 (26.8)**	207 (21.0)	**463 (24.9)**	3201 (18.6)
Ischemic heart disease	4 (0.8)	1 (0.4)	0 (0.0)	24 (1.0)	**409 (25.9)**	**226 (22.9)**	**463 (24.9)**	3174 (18.5)
Cancer	5 (1.0)	3 (1.1)	1 (0.5)	22 (0.9)	**87 (5.5)**	**52 (5.3)**	**111 (6.0)**	610 (3.5)
COPD	1 (0.2)	1 (0.4)	2 (1.0)	14 (0.6)	**185 (11.7)**	103 (10.4)	**227 (12.2)**	1504 (8.7)
Liver disease	3 (0.6)	1 (0.4)	0 (0.0)	17 (0.7)	184 (11.6)	97 (9.8)	**249 (13.4)**	1770 (10.3)
**Country**								
Argentina	185	97	100	1021				
Colombia	177	108	55	822				
Ecuador	158	70	51	629				
United States					1581	988	1857	17,198

*P*-values for univariate comparison of died versus survived (reference) are shown with bold font representing *p* < 0.05. All patient characteristics presented as of the COVID-19 presentation date

**Table 4:** Clinical profiles of HD patients who died or survived any time after COVID-19 presentation in LatAm

	**LatAm**					
	**Most Recent value 0 to 14 days prior to COVID-19 symptoms**		**Most Recent value > 14 days prior to COVID-19 symptoms**		**Delta**	
	**Mean ± SD or N (%)**		**Mean ± SD or N (%)**		**Mean ± SD or N (%)**	
**Parameter**	**Died any time**	**Survived**	**Died any time**	**Survived**	**Died any time**	**Survived**
**Laboratories**						
Albumin	**3.63 ± 0.5**	3.82 ± 0.5	**3.79 ± 0.5**	3.92 ± 0.4	-0.07 ± 0.4	-0.03 ± 0.3
Calcium	8.54 ± 0.8	8.56 ± 0.8	8.66 ± 0.7	8.69 ± 0.8	-0.12 ± 0.6	-0.09 ± 0.6
Corrected calcium	8.74 ± 0.7	8.65 ± 0.7	**8.82 ± 0.7**	8.73 ± 0.7	-0.06 ± 0.6	-0.03 ± 0.6
Creatinine	**7.85 ± 2.7**	8.6 ± 2.8	**7.84 ± 2.6**	8.46 ± 2.6	**-0.05 ± 1.8**	0.21 ± 1.7
Ferritin	641.63 ± 362.2	640.58 ± 392.9	662.52 ± 391.7	645.65 ± 375	29.33 ± 286.9	41.76 ± 296.1
Hgb	**10.87 ± 1.9**	11.08 ± 1.8	**10.98 ± 1.9**	11.22 ± 1.7	-0.12 ± 1.3	-0.05 ± 1.2
Lymphocytes	**21.14 ± 9**	24.04 ± 8.7	**22.96 ± 8.4**	24.81 ± 9	-2.14 ± 8.1	-1.08 ± 7.1
Neutrophils	**64.77 ± 12.4**	61.52 ± 11.2	**62.8 ± 10.7**	60.74 ± 11.3	2.81 ± 10.8	1.41 ± 10.1
Phosphate	**4.36 ± 1.5**	4.57 ± 1.6	**4.39 ± 1.5**	4.54 ± 1.4	-0.04 ± 1.3	-0.05 ± 1.2
Platelets	**63,182.61 ± 98,067.2**	81,159.84 ± 102,855.6	**75,393.6 ± 103,458.4**	82,138.8 ± 106,112.2	-3052.9 ± 32,591.7	-2888.8 ± 29,308.2
Parathyroid hormone	**353.2 ± 311.5**	443.5 ± 367.4	**410.1 ± 372**	448.9 ± 391.1	**-27.6 ± 211.9**	24.1 ± 215.1
Transferrin saturation	**26.02 ± 13.8**	28.31 ± 14.6	**29.25 ± 14.2**	31.27 ± 14.7	-4.96 ± 15.1	-2.58 ± 13.9
White blood cell count	**2231.18 ± 3487.6**	2653.74 ± 3419.9	2664.44 ± 3664.5	2697.14 ± 3575.9	-24.74 ± 1522.9	-152.61 ± 1482.9
**Vital signs**						
Pre-HD SBP	134.7 ± 25.4	135.65 ± 25.2	137.94 ± 25.3	138.18 ± 24.4	-2.89 ± 23.8	-2.14 ± 22.1
Post-HD SBP	129.17 ± 24.9	129.8 ± 24.4	128.91 ± 23.7	128.95 ± 23.3	0.86 ± 22.5	1.24 ± 19.9
Pre-HD DBP	**69.45 ± 12.9**	70.89 ± 13.1	**69.6 ± 12.7**	72.05 ± 13.4	-0.19 ± 13.9	-1.16 ± 13.4
Post-HD DBP	68.27 ± 11.4	68.85 ± 12.1	67.91 ± 11.7	68.66 ± 12.1	0.48 ± 12.6	0.34 ± 123
Pre-HD pulse	77.2 ± 10.6	77.61 ± 10.2	76.15 ± 10	76.76 ± 9.4	0.65 ± 11.4	0.88 ± 10.4
Post-HD pulse	76.75 ± 10.4	77.04 ± 9.6	**75.19 ± 8.7**	75.98 ± 9.1	1.25 ± 11.3	1.12 ± 10.3
Pre-HD temperature	36.39 ± 0.5	36.35 ± 0.5	36.6 ± 0.6	36.34 ± 0.6	-0.23 ± 0.6	-0.29 ± 0.6
Post-HD temperature	36.18 ± 0.4	36.18 ± 0.4	36.12 ± 0.3	36.13 ± 0.3	0.07 ± 0.4	0.06 ± 0.5
**Weights**						
Pre-HD weight	73.14 ± 19.6	73.28 ± 17.7	73.42 ± 19.5	73.75 ± 17.5	-0.67 ± 3.0	-0.43 ± 2.9
Post-HD weight	71.37 ± 19.3	71.42 ± 17.6	71.43 ± 19	71.45 ± 17.2	-0.39 ± 3.1	-0.02 ± 5.4
IDWG	**1.74 ± 1.5**	1.94 ± 1.5	**2.08 ± 1.4**	2.24 ± 1.3	-0.35 ± 1.8	-0.28 ± 1.7
Dry weight	71.52 ± 18.8	71.1 ± 17.4	71.22 ± 18.8	71.13 ± 17.2	-0.1 ± 1.5	-0.04 ± 0.7

*P*-values for univariate comparison of died versus survived (reference) are shown with bold font representing *p* < 0.05. Albumin and hemoglobin (Hgb) are reported in units of g/dL. Calcium, corrected calcium, creatinine, and phosphate are reported in units of mg/dL. Ferritin is reported in unit of ng/mL. Parathyroid hormone is reported in the unit of pg/mL. Lymphocytes, neutrophils, and transferrin saturation (TSAT) are reported in unit of percent (%). Platelets and white blood cell counts are reported in unit of mm^3. Systolic/diastolic blood pressures (SBP/DBP) are reported in units of mmHg. Pulse is reported in unit of BPM. Temperature is reported in unit of Celsius. Weights are reported in unit of Kg

**Table 5:** Clinical profiles of HD patients who died or survived any time after COVID-19 presentation in North America

	**North America**					
	**Most Recent value 0 to 14 days prior to COVID-19 symptoms**		**Most Recent value > 14 days prior to COVID-19 symptoms**		**Delta**	
	**Mean ± SD or N (%)**		**Mean ± SD or N (%)**		**Mean ± SD or N (%)**	
**Parameter**	**Died any time**	**Survived**	**Died any time**	**Survived**	**Died any time**	**Survived**
**Laboratories**						
Albumin	**3.47 ± 0.53**	3.70 ± 0.44	**3.55 ± 0.50**	3.75 ± 0.42	**-0.06 ± 0.31**	-0.04 ± 0.28
Calcium	8.71 ± 0.76	8.72 ± 0.73	**8.80 ± 0.69**	8.84 ± 0.68	**-0.08 ± 0.61**	-0.12 ± 0.60
Corrected calcium	**9.14 ± 0.71**	8.97 ± 0.69	**9.16 ± 0.67**	9.04 ± 0.68	**-0.03 ± 0.59**	-0.08 ± 0.57
Creatinine	**7.37 ± 2.77**	8.40 ± 3.24	**7.42 ± 2.70**	8.35 ± 3.06	**0.07 ± 1.47**	0.22 ± 1.56
Ferritin	961.65 ± 436.42	963.78 ± 433.18	**987.57 ± 418.42**	955.72 ± 418.56	**24.20 ± 345.33**	62.77 ± 346.94
Hgb	**10.36 ± 1.41**	10.56 ± 1.34	**10.41 ± 1.37**	10.65 ± 1.29	-0.05 ± 0.96	-0.06 ± 0.90
Lymphocytes	**17.07 ± 8.77**	19.26 ± 8.26	**18.16 ± 8.27**	20.21 ± 7.99	-0.99 ± 6.79	-1.07 ± 6.50
Neutrophils	**69.89 ± 10.64**	67.35 ± 9.97	**68.38 ± 9.95**	66.17 ± 9.58	1.36 ± 8.81	1.25 ± 8.53
Phosphate	**5.09 ± 1.73**	5.37 ± 1.72	**5.12 ± 1.70**	5.38 ± 1.68	-0.09 ± 1.53	-0.07 ± 1.49
Platelets	191.69 ± 87.18	191.99 ± 78.34	**195.40 ± 86.05**	199.80 ± 76.65	**-5.03 ± 59.92**	-9.73 ± 55.50
Parathyroid hormone	**401.03 ± 325.02**	456.62 ± 325.89	**392.28 ± 290.29**	427.66 ± 304.70	**-17.36 ± 236.18**	1.38 ± 232.09
Transferrin saturation	29.64 ± 14.17	29.59 ± 14.19	**31.82 ± 14.06**	32.92 ± 14.07	**-2.03 ± 15.69**	-2.99 ± 16.35)
White blood cell count	**7.01 ± 2.98**	6.57 ± 2.66	**7.18 ± 3.34**	6.92 ± 2.54	**-0.17 ± 2.58**	-0.41 ± 2.34
**Vital signs**						
Pre-HD SBP	**139.59 ± 27.88**	145.83 ± 27.24	**143.18 ± 27.12**	148.60 ± 26.37	-3.59 ± 27.49	-2.70 ± 27.23
Post-HD SBP	**140.45 ± 26.40**	144.50 ± 25.97	**138.17 ± 24.52**	140.80 ± 24.64	**2.21 ± 26.34**	3.76 ± 25.72
Pre-HD DBP	**71.17 ± 15.56**	75.65 ± 15.98	**72.53 ± 15.16**	77.04 ± 15.74	-1.32 ± 16.05	-1.35 ± 15.89
Post-HD DBP	**71.60 ± 14.89**	75.10 ± 14.92	**70.79 ± 14.02**	73.91 ± 14.23	0.83 ± 15.47	1.20 ± 15.08
Pre-HD pulse	80.15 ± 14.75	80.52 ± 13.73	**77.57 ± 13.65**	78.34 ± 12.86	**2.66 ± 13.31**	2.18 ± 12.13
Post-HD pulse	**80.48 ± 15.06**	79.66 ± 13.95	76.21 ± 13.08	75.88 ± 12.32	**4.35 ± 14.96**	3.83 ± 13.42
Pre-HD temperature	**36.56 ± 0.54**	36.59 ± 0.55	36.40 ± 0.43	36.41 ± 0.43	0.17 ± 0.63	0.19 ± 0.62
Post-HD temperature	**36.56 ± 0.58**	36.62 ± 0.59	**36.39 ± 0.40**	36.42 ± 0.39	**0.18 ± 0.64**	0.20 ± 0.65
**Weights**						
Pre-HD weight	**82.93 ± 24.56**	86.15 ± 24.73	**83.64 ± 24.52**	87.02 ± 24.81	-0.81 ± 2.77	-0.88 ± 2.73
Post-HD weight	**81.26 ± 24.10**	84.32 ± 24.31	**81.58 ± 23.99**	84.74 ± 24.31	-0.41 ± 2.67	-0.44 ± 2.13
IDWG	**1.90 ± 1.58**	1.96 ± 1.56	**2.23 ± 1.60**	2.37 ± 1.48	**-0.35 ± 1.87**	-0.43 ± 1.75
Dry weight	**81.08 ± 23.94**	84.34 ± 24.24	**81.27 ± 23.82**	84.43 ± 24.23	**-0.31 ± 1.93**	-0.15 ± 1.77

*P-*values for univariate comparison of died versus survived (reference) are shown with bold font representing *p* < 0.05. Albumin and hemoglobin (Hgb) are reported in units of g/dL. Calcium, corrected calcium, creatinine, and phosphate are reported in units of mg/dL. Ferritin is reported in unit of ng/mL. Parathyroid hormone is reported in the unit of pg/mL. Lymphocytes, neutrophils, and transferrin saturation (TSAT) are reported in unit of percent (%). Platelets and white blood cell counts are reported in unit of 10^3/µL. Systolic/diastolic blood pressures (SBP/DBP) are reported in units of mmHg. Pulse is reported in unit of BPM. Temperature is reported in unit of Celsius. Weights are reported in unit of Kg

**Table 6:** Clinical profiles of HD patients who died or survived 0 to 14, 15 to 30, and > 30 days after COVID-19 presentation in LatAm

	**LatAm**											
	**Most Recent value 0 to 14 days prior to COVID symptoms**					**Most Recent value > 14 days prior to COVID symptoms**				**Delta**		
	**Mean ± SD or N (%)**					**Mean ± SD or N (%)**				**Mean ± SD or N (%)**		
**Parameter**	**Died 0–14 days**	**Died 15–30 days**	**Died > 30 days**	**Survived**	**Died 0–14 days**	**Died 15–30 days**	**Died > 30 days**	**Survived**	**Died 0–14 days**	**Died 15–30 days**	**Died > 30 days**	**Survived**
**Laboratories**												
Albumin	**3.7 ± 0.6**	**3.65 ± 0.5**	**3.63 ± 0.4**	3.82 ± 0.5	**3.77 ± 0.5**	**3.82 ± 0.5**	**3.8 ± 0.5**	3.92 ± 0.4	-0.04 ± 0.4	-0.1 ± 0.4	-0.12 ± 0.3	-0.03 ± 0.3
Calcium	8.54 ± 0.8	8.52 ± 0.8	8.55 ± 0.8	8.56 ± 0.8	8.7 ± 0.7	8.63 ± 0.8	8.66 ± 0.7	8.69 ± 0.8	-0.17 ± 0.6	-0.1 ± 0.6	-0.02 ± 0.7	-0.09 ± 0.6
Corrected calcium	8.73 ± 0.7	8.7 ± 0.9	8.84 ± 0.7	8.65 ± 0.7	**8.85** ± 0.8	8.75 ± 0.7	**8.83** ± 0.7	8.73 ± 0.7	-0.06 ± 0.7	-0.16 ± 0.6	0.09 ± 0.7	-0.03 ± 0.6
Creatinine	**7.85 ± 2.6**	**7.86 ± 2.8**	**7.86 ± 3**	8.6 ± 2.8	**7.91 ± 2.5**	**7.87 ± 2.8**	**7.63 ± 2.6**	8.46 ± 2.6	-0.07 ± 1.9	0.08 ± 1.5	**-0.24 ± 2.2**	0.21 ± 1.7
Ferritin	691.22 ± 344	**504.23 ± 328.1**	707.11 ± 428.5	640.58 ± 392.9	**676.99 ± 402.2**	621.9 ± 361.6	681.89 ± 402.4	645.65 ± 375	24.66 ± 306.7	-24.52 ± 257.7	139.38 ± 258	41.76 ± 296.1
Hgb	**10.85 ± 1.9**	11.12 ± 1.8	**10.61 ± 2.2**	11.08 ± 1.8	**11 ± 1.8**	11.15 ± 1.9	**10.67 ± 2.2**	11.22 ± 1.7	-0.17 ± 1.4	-0.15 ± 1.2	0.03 ± 1.4	-0.05 ± 1.2
Lymphocytes	**21.39 ± 9.1**	**21.21 ± 8.6**	**20.37 ± 9.5**	24.04 ± 8.7	**23.3 ± 8.5**	**23.16 ± 8**	**21.7 ± 8.8**	24.81 ± 9	**-2.79 ± 7.9**	-1.34 ± 7.7	-1.55 ± 9.2	-1.08 ± 7.1
Neutrophils	**64.42 ± 12.3**	**64.15 ± 12.3**	**66.58 ± 12.8**	61.52 ± 11.2	**62.31 ± 10.7**	**62.49 ± 10.4**	**64.68 ± 11**	60.74 ± 11.3	**4 ± 10.3**	1.65 ± 10.2	1.31 ± 12.6	1.41 ± 10.1
Phosphate	**4.31 ± 1.5**	4.56 ± 1.6	**4.23 ± 1.5**	4.57 ± 1.6	**4.38 ± 1.5**	4.41 ± 1.4	4.42 ± 1.3	4.54 ± 1.4	-0.04 ± 1.3	0.09 ± 1.2	-0.2 ± 1.4	-0.05 ± 1.2
Platelets	**53,584.6 ± 95,054.3**	66,566.43 ± 96,522.7	80,784.93 ± 105,396.4	81,159.84 ± 102,855.6	**73,057.12 ± 105,585**	**66,788.54 ± 97,783.3**	92,688.7 ± 104,532.8	82,138.8 ± 106,112.2	-2706.11 ± 24,275	-4489.33 ± 35,190.8	-1914.45 ± 43,479.7	-2888.8 ± 29,308.2
Parathyroid hormone	364.45 ± 329.3	361.3 ± 308.1	**288.23 ± 233.2**	443.5 ± 367.4	414.57 ± 370.2	**398.49 ± 363.5**	414.61 ± 389.6	448.9 ± 391.1	-18.72 ± 238	-24.19 ± 157.8	**-70.46** ± **202.7**	24.1 ± 215.1
Transferrin saturation	27.42 ± 13.7	**24.04 ± 13.5**	24.08 ± 14.7	28.31 ± 14.6	29.53 ± 14.8	**29.43 ± 14.1**	**28.31 ± 12.7**	31.27 ± 14.7	-4.75 ± 16.7	-4.45 ± 12.9	-6.68 ± 13.1	-2.58 ± 13.9
White blood cell count	**1879.38 ± 3296.9**	2436.48 ± 3554.4	2767.92 ± 3772.3	2653.74 ± 3419.9	2602.38 ± 3766.6	**2366.94 ± 3415.6**	**3221.25 ± 3705.9**	2697.14 ± 3575.9	-3.71 ± 1415.3	-46.11 ± 1576	-42.21 ± 1688.8	-152.61 ± 1482.9
**Vital signs**												
Pre-HD SBP	134.67 ± 25.3	134 ± 24.7	135.48 ± 26.4	135.65 ± 25.2	136.86 ± 24.9	138.81 ± 25.3	139.4 ± 26.3	138.18 ± 24.4	-2.26 ± 23.5	-4.68 ± 23	-2.28 ± 25.6	-2.14 ± 22.1
Post-HD SBP	128.84 ± 24.6	129.33 ± 24.2	129.7 ± 26.4	129.8 ± 24.4	127.87 ± 23.9	129.44 ± 23.3	130.63 ± 23.5	128.95 ± 23.3	1.41 ± 23.2	0.86 ± 20.8	-0.26 ± 23	1.24 ± 19.9
Pre-HD DBP	**68.99 ± 13.1**	70.72 ± 12.9	69.11 ± 12.6	70.89 ± 13.1	**68.32 ± 12.2**	70.89 ± 12.7	71.01 ± 13.7	72.05 ± 13.4	**0.55 ± 14.7**	-0.14 ± 12.5	-1.75 ± 13.4	-1.16 ± 13.4
Post-HD DBP	**67.1 ± 10.8**	70.27 ± 11.1	68.64 ± 12.6	68.85 ± 12.1	**67.36 ± 12.2**	68.91 ± 10.9	68.05 ± 11.5	68.66 ± 12.1	-0.07 ± 12.9	1.67 ± 12.5	0.34 ± 11.9	0.34 ± 123
Pre-HD pulse	76.85 ± 10.8	77.74 ± 11.1	77.39 ± 9.7	77.61 ± 10.2	**75.64 ± 9.9**	76.46 ± 9.2	76.96 ± 11.2	76.76 ± 9.4	0.85 ± 11.4	0.6 ± 10.1	0.31 ± 12.5	0.88 ± 10.4
Post-HD pulse	76.7 ± 10.9	76.16 ± 8.7	77.47 ± 10.9	77.04 ± 9.6	**74.89 ± 8.2**	75.92 ± 9.1	75.08 ± 9.4	75.98 ± 9.1	1.6 ± 12	-0.29 ± 10	2.17 ± 11.2	1.12 ± 10.3
Pre-HD temperature	36.37 ± 0.4	**36.51** ± 0.7	36.31 ± 0.5	36.35 ± 0.5	36.59 ± 0.5	36.63 ± 0.6	36.59 ± 0.6	36.34 ± 0.6	-0.25 ± 0.5	-0.15 ± 0.7	-0.28 ± 0.6	-0.29 ± 0.6
Post-HD temperature	36.15 ± 0.4	**36.28** ± 0.5	36.12 ± 0.4	36.18 ± 0.4	36.11 ± 0.3	36.16 ± 0.3	36.1 ± 0.3	36.13 ± 0.3	0.04 ± 0.4	0.13 ± 0.6	0.05 ± 0.4	0.06 ± 0.5
**Weights**												
Pre-HD weight	74.54 ± 20	74.11 ± 22.1	**69.25 ± 15.1**	73.28 ± 17.7	74.99 ± 19.6	73.91 ± 22	**69.5 ± 15.7**	73.75 ± 17.5	**-0.78 ± 2.7**	-0.51 ± 2.8	-0.6 ± 3.6	-0.43 ± 2.9
Post-HD weight	72.8 ± 19.6	72.34 ± 21.7	**67.4 ± 15**	71.42 ± 17.6	73.17 ± 19	71.89 ± 21.6	67.16 ± 15.5	71.45 ± 17.2	**-0.6 ± 3.6**	-0.28 ± 2.4	-0.08 ± 2.7	-0.02 ± 5.4
IDWG	**1.55 ± 1.4**	1.9 ± 1.6	1.97 ± 1.5	1.94 ± 1.5	**2.07 ± 1.5**	2.11 ± 1.5	2.09 ± 1.2	2.24 ± 1.3	-0.56 ± 1.8	-0.21 ± 1.8	-0.07 ± 1.7	-0.28 ± 1.7
Dry weight	73.1 ± 18.9	72.4 ± 21.6	**67.35 ± 14.6**	71.1 ± 17.4	**72.87 ± 18.7**	71.55 ± 21.6	**67.32 ± 15**	71.13 ± 17.2	-0.16 ± 0.7	**0.09 ± 0.7**	**-0.19 ± 2.8**	-0.04 ± 0.7

*P*-values for univariate comparison of died versus survived (reference) are shown with bold font representing *p* < 0.05. Albumin and hemoglobin (Hgb) are reported in units of g/dL. Calcium, corrected calcium, creatinine, and phosphate are reported in units of mg/dL. Ferritin is reported in unit of ng/mL. Parathyroid hormone is reported in the unit of pg/mL. Lymphocytes, neutrophils, and transferrin saturation (TSAT) are reported in unit of percent (%). Platelets and white blood cell counts are reported in unit of mm^3. Systolic/diastolic blood pressures (SBP/DBP) are reported in units of mmHg. Pulse is reported in unit of BPM. Temperature is reported in unit of Celsius. Weights are reported in unit of Kg

**Table 7:** Clinical profiles of HD patients who died or survived 0 to 14, 15 to 30, and > 30 days after COVID-19 presentation in North America

	**North America**													
	**Most Recent value 0 to 14 days prior to COVID symptoms**					**Most Recent value > 14 days prior to COVID symptoms**					**Delta**			
	**Mean ± SD or N (%)**					**Mean ± SD or N (%)**					**Mean ± SD or N (%)**			
**Parameter**	**Died 0–14 days**	**Died 15–30 days**	**Died > 30 days**	**Survived**	**Died 0–14 days**		**Died 15–30 days**	**Died > 30 days**	**Survived**	**Died 0–14 days**		**Died 15–30 days**	**Died > 30 days**	**Survived**
**Laboratories**														
Albumin	**3.47 ± 0.55**	**3.55 ± 0.52**	**3.42 ± 0.52**	3.70 ± 0.44	**3.58 ± 0.51**		**3.62 ± 0.48**	**3.48 ± 0.50**	3.75 ± 0.42	**-0.07 ± 0.31**		-0.06 ± 0.31	-0.05 ± 0.31	-0.04 ± 0.28
Calcium	8.70 ± 0.74	8.74 ± 0.72	8.70 ± 0.80	8.72 ± 0.73	8.82 ± 0.66		**8.79 ± 0.65**	**8.78 ± 0.72**	8.84 ± 0.68	-0.11 ± 0.58		**-0.06 ± 0.60**	**-0.07 ± 0.64**	-0.12 ± 0.60
Corrected calcium	**9.14 ± 0.68**	**9.10 ± 0.70**	**9.16 ± 0.74**	8.97 ± 0.69	**9.16 ± 0.66**		**9.10 ± 0.65**	**9.19 ± 0.68**	9.04 ± 0.68	-0.04 ± 0.55		**-0.01 ± 0.61**	**-0.03 ± 0.62**	-0.08 ± 0.57
Creatinine	**7.48 ± 2.89**	**7.74 ± 2.80**	**7.07 ± 2.63**	8.40 ± 3.24	**7.51 ± 2.72**		**7.77 ± 2.82**	**7.16 ± 2.59**	8.35 ± 3.06	0.12 ± 1.46		0.12 ± 1.46	**0.00 ± 1.47**	0.22 ± 1.56
Ferritin	956.16 ± 436.70	985.37 ± 441.67	952.81 ± 433.73	963.78 ± 433.18	**988.30 ± 411.87**		**1006.37 ± 418.79**	976.56 ± 423.73	955.72 ± 418.56	41.40 ± 339.42		23.93 ± 363.71	**10.77 ± 339.47**	62.77 ± 346.94
Hgb	**10.40 ± 1.35**	**10.48 ± 1.44**	**10.25 ± 1.44**	10.56 ± 1.34	**10.46 ± 1.31**		10.59 ± 1.39	**10.27 ± 1.39**	10.65 ± 1.29	-0.03 ± 0.92		-0.09 ± 0.91	-0.03 ± 1.01	-0.06 ± 0.90
Lymphocytes	**16.76 ± 9.28**	**17.08 ± 8.03**	**17.32 ± 8.78**	19.26 ± 8.26	**18.23 ± 8.81**		**18.52 ± 7.47**	**17.91 ± 8.20**	20.21 ± 7.99	**-1.60 ± 6.90**		-1.32 ± 6.66	**-0.31 ± 6.73**	-1.07 ± 6.50
Neutrophils	**70.27 ± 10.96**	**69.99 ± 10.10**	**69.52 ± 10.70**	67.35 ± 9.97	**68.41 ± 10.34**		**68.05 ± 9.10**	**68.55 ± 10.05**	66.17 ± 9.58	1.83 ± 8.92		1.72 ± 8.44	0.76 ± 8.90	1.25 ± 8.53
Phosphate	**5.07 ± 1.73**	**5.07 ± 1.59**	**5.14 ± 1.80**	5.37 ± 1.72	**5.03 ± 1.68**		**5.20 ± 1.74**	**5.15 ± 1.70**	5.38 ± 1.68	-0.06 ± 1.43		-0.16 ± 1.50	-0.09 ± 1.63	-0.07 ± 1.49
Platelets	195.83 ± 90.83	187.57 ± 78.68	190.68 ± 88.67	191.99 ± 78.34	196.95 ± 87.36		**192.81 ± 78.38**	**195.43 ± 88.79**	199.80 ± 76.65	-6.70 ± 60.57		**-2.55 ± 56.37**	-5.11 ± 61.36	-9.73 ± 55.50
Parathyroid hormone	**392.91 ± 308.39**	**408.23 ± 329.49**	**403.69 ± 336.24**	456.62 ± 325.89	**384.75 ± 279.71**		**403.60 ± 293.19**	**392.67 ± 297.63**	427.66 ± 304.70	**-22.71 ± 224.93**		-23.40 ± 241.05	-9.38 ± 242.53	1.38 ± 232.09
Transferrin saturation	29.39 ± 14.71	30.12 ± 13.42	29.57 ± 14.14	29.59 ± 14.19	**31.79 ± 13.93**		32.86 ± 13.87	**31.29 ± 14.26**	32.92 ± 14.07	-1.90 ± 15.18		-2.42 ± 14.41	-1.91 ± 16.79	-2.99 ± 16.35
White blood cell count	**7.17 ± 3.22**	**6.88 ± 2.99**	**6.95 ± 2.75**	6.57 ± 2.66	**7.33 ± 3.42**		7.04 ± 2.80	**7.13 ± 3.54**	6.92 ± 2.54	**-0.06 ± 2.54**		**-0.17 ± 2.91**	-0.26 ± 2.39	-0.41 ± 2.34
**Vital signs**														
Pre-HD SBP	**138.0 ± 28.1**	**141.9 ± 27.4**	**139.7 ± 27.8**	145.8 ± 27.2	**72.4 ± 15.5**		**73.0 ± 14.2**	**72.4 ± 15.4**	77.0 ± 15.7	**-4.5 ± 27.6**		-3.1 ± 27.3	-3.0 ± 27.5	-2.7 ± 27.2
Post-HD SBP	**140.6 ± 27.1**	**140.7 ± 26.3**	**140.2 ± 25.8**	144.5 ± 26.0	**70.5 ± 13.8**		**71.1 ± 13.2**	**70.8 ± 14.6**	73.9 ± 14.2	3.3 ± 26.4		**2.0 ± 26.6**	**1.4 ± 26.1**	3.8 ± 25.7
Pre-HD DBP	**70.5 ± 15.5**	**72.1 ± 15.4**	**71.3 ± 15.6**	75.7 ± 16.0	**142.4 ± 27.4**		**145.2 ± 26.7**	**142.8 ± 27.0**	148.6 ± 26.4	-2.0 ± 16.5		-0.9 ± 15.4	**-1.0 ± 16.1**	-1.4 ± 15.9
Post-HD DBP	**72.1 ± 15.2**	**71.5 ± 14.6**	**71.2 ± 14.8**	75.1 ± 14.9	**137.3 ± 24.6**		**138.7 ± 24.0**	**138.7 ± 24.7**	140.8 ± 24.6	1.6 ± 15.9		0.4 ± 14.8	**0.4 ± 15.5**	1.20 ± 15.1
Pre-HD pulse	**81.08 ± 14.96**	80.80 ± 14.63	**78.98 ± 14.55**	80.52 ± 13.73	**77.13 ± 13.13**		77.93 ± 13.61	77.75 ± 14.10	78.34 ± 12.86	**3.93 ± 13.76**		2.88 ± 13.26	**1.44 ± 12.84**	2.18 ± 12.13
Post-HD pulse	**81.83 ± 15.00**	**81.01 ± 15.07**	79.01 ± 14.98	79.66 ± 13.95	76.32 ± 12.85		76.08 ± 12.67	76.18 ± 13.50	75.88 ± 12.32	**5.49 ± 15.37**		**4.91 ± 14.91**	**3.06 ± 14.54**	3.83 ± 13.42
Pre-HD temperature	36.58 ± 0.58	36.56 ± 0.53	**36.55 ± 0.52**	36.59 ± 0.55	**36.38 ± 0.43**		36.40 ± 0.42	36.41 ± 0.43	36.41 ± 0.43	0.20 ± 0.66		0.16 ± 0.61	**0.14 ± 0.60**	0.19 ± 0.62
Post-HD temperature	36.60 ± 0.62	**36.56 ± 0.58**	**36.52 ± 0.53**	36.62 ± 0.59	**36.39 ± 0.40**		**36.38 ± 0.39**	**36.39 ± 0.40**	36.42 ± 0.39	0.22 ± 0.68		0.19 ± 0.65	**0.14 ± 0.60**	0.20 ± 0.65
**Weights**														
Pre-HD weight	**84.39 ± 25.27**	**84.41 ± 24.45**	**80.85 ± 23.86**	86.15 ± 24.73	**85.19 ± 25.32**		**85.00 ± 24.09**	**81.56 ± 23.90**	87.02 ± 24.81	-0.92 ± 2.79		-0.78 ± 2.78	**-0.72 ± 2.74**	-0.88 ± 2.73
Post-HD weight	**82.82 ± 24.81**	**82.70 ± 23.96**	**79.11 ± 23.40**	84.32 ± 24.31	**83.09 ± 24.82**		**82.92 ± 23.51**	**79.55 ± 23.38**	84.74 ± 24.31	-0.39 ± 3.07		-0.41 ± 2.39	-0.44 ± 2.42	-0.44 ± 2.13
IDWG	**1.79 ± 1.47**	1.95 ± 1.67	1.96 ± 1.61	1.96 ± 1.56	**2.19 ± 1.62**		2.28 ± 1.52	**2.23 ± 1.62**	2.37 ± 1.48	-0.42 ± 1.75		-0.35 ± 1.90	**-0.28 ± 1.94**	-0.43 ± 1.75
Dry weight	**82.70 ± 24.69**	**82.53 ± 23.77**	**78.86 ± 23.20**	84.34 ± 24.24	**82.86 ± 24.60**		**82.57 ± 23.24**	**79.19 ± 23.30**	84.43 ± 24.23	**-0.28 ± 1.88**		**-0.29 ± 1.98**	**-0.34 ± 1.94**	-0.15 ± 1.77

*P*-values for univariate comparison of died versus survived (reference) are shown with bold font representing *p* < 0.05. Albumin and hemoglobin (Hgb) are reported in units of g/dL. Calcium, corrected calcium, creatinine, and phosphate are reported in units of mg/dL. Ferritin is reported in unit of ng/mL. Parathyroid hormone is reported in the unit of pg/mL. Lymphocytes, neutrophils, and transferrin saturation (TSAT) are reported in unit of percent (%). Platelets and white blood cell counts are reported in unit of 10^3/µL. Systolic/diastolic blood pressures (SBP/DBP) are reported in units of mmHg. Pulse is reported in unit of BPM. Temperature is reported in unit of Celsius. Weights are reported in unit of Kg

### Model performance

The machine learning models constructed to establish the predictors of mortality in COVID-19 were found to have suitable performance in prediction of the outcome of death in both regions overall, as well as in the predefined shorter timeframe after COVID-19 presentation (Table 8). The AUC for the model’s classification of death at any time after COVID-19 presentation was 0.76 in LatAm cohort and 0.79 in North America cohort, the balanced accuracy was 71% in the LatAm cohort and 70% in North America cohort, and the AUPRC was 0.21 in LatAm cohort and 0.52 in North America cohort.

**Table 8:** Performance of machine learning models in predicting death after COVID-19 presentation

		**LatAm** ***Time after COVID-19***				**North America** ***Time after COVID-19***				
**Metric**	**Dataset**	*Any time*	*0–14 days*	*15–30 days*	> *30 days*		*Any time*	*0–14 days*	*15–30 days*	> *30 days*
***Area under the curve***	*Training*	0.999	0.959	0.997	0.996		0.935	0.986	0.979	0.954
	*Validation*	0.783	0.755	0.755	0.739		0.779	0.812	0.790	0.802
	*Testing*	0.763	0.746	0.728	0.809		0.790	0.828	0.788	0.813
***Balanced accuracy***	*Training*	96.5	81.9	95.1	91.9		80.9	89.6	86.9	84.1
	*Validation*	70.2	66.7	73.3	70.0		68.1	72.4	70.2	70.0
	*Testing*	70.8	66.1	66.0	74.6		69.8	73.9	71.2	71.2
**Area under precision-recall curve**	*Testing*	0.210	0.375	0.062	0.040		0.515	0.298	0.233	0.356

The area under the curve (AUC) and area under precision-recall curve (AUPRC) are presented on a scale from 0 (lowest) to 1 (highest). Chance equals a value of 0.5 for AUC. Chance equals the fraction of positive cases in each regional group for each model for AUPRC (i.e., the number of patients who died in each group divided by the total number of patients in each group). Balanced accuracy is presented as a percentage

Relatively consistent AUCs (ranging from 0.73 to 0.83) and balanced accuracy (ranging from 66 to 75%) were found across models in predefined timeframes 0 to 14, 15 to 30, and > 30 days after COVID-19 for both regions. Considering the AUPRC, the model was found to have suitable performance in classification of shorter-term death events within 0 to 14 days after COVID-19 presentation in both the LatAm cohort (AUPRC = 0.38) and North America cohort (AUPRC = 0.30). Although the AUPRC showed suitable performance in the North America cohort for classification of the risk of death 15 to 30 days (AUPRC = 0.23) and > 30 days (AUPRC = 0.36) after COVID-19, it showed poor performance in prediction of intermediate- (AUPRC = 0.06) and longer-term (AUPRC = 0.04) outcomes in the LatAm cohort.

### Predictors of death any time after COVID-19

**Fig. 4:**
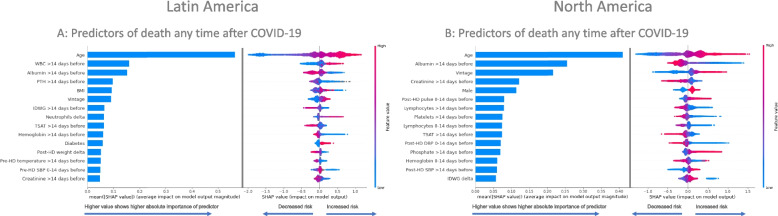
Top 15 predictors of death any time after COVID-19 presentation in descending order for the Latin America (**A**) and North America (**B**) cohorts. Bar plot in the left panels show the mean absolute SHAP values that estimate the average size of each variable’s contribution to predicting the outcome on the x-axis (calculated from the average absolute value for all patients). SHAP value plots in the right panels show the size and direction (more positive = higher risk or more negative = lower risk) of each variable’s influence on the outcome for each unique patient on the x-axis, with warmer colors representing higher observed values for that measurement, cooler colors indicating lower values for that measurement, and gray representing a missing value for that measurement. SHAP values are presented in the unit of log odds (i.e. logarithm of the odds ratio)

We estimated the importance of each predictor variable with SHAP values and found the top three predictors of death any time after COVID-19 presentation in the LatAm cohort were older age, higher WBC counts historically (i.e. > 14 days prior to COVID-19 presentation), and lower albumin levels historically; in North America, the top three predictors included older age, lower albumin levels historically, and longer dialysis vintage. In Fig. 4, the bar charts on the left side of each panel show the mean absolute SHAP values that represent the magnitude of importance for each variable in log odds; these are shown in descending order of importance for the top 15 predictors. The SHAP value plots on the right of each panel further show the degree and direction of the effect for each variable on each unique patient’s prediction. The SHAP value plots denote a dot that corresponds to each patient and the dot’s position on the x-axis (positive or negative) represents the magnitude of that variable’s effect on the risk prediction for that unique patient. The color of each dot on the SHAP value plots indicate how large/high or small/low the value is for that variable in that unique patient’s prediction. For an example with the top predictor of age, the mean SHAP values show age has a high magnitude of importance as compared to other variables and the SHAP value plots show more positive SHAP values for dots that had warmer colors (representing increasing age with the warmer the color and increasing risk based on how positive the value is), and more negative SHAP values for dots that had cooler colors (representing younger age with the cooler the color and decreasing risk based on how negative the value is). Age showed the largest contribution to the risk of death after COVID-19; however, many variables had a high magnitude considering the log odds values and the distributions of risks in SHAP value plots.

**Fig. 5:**
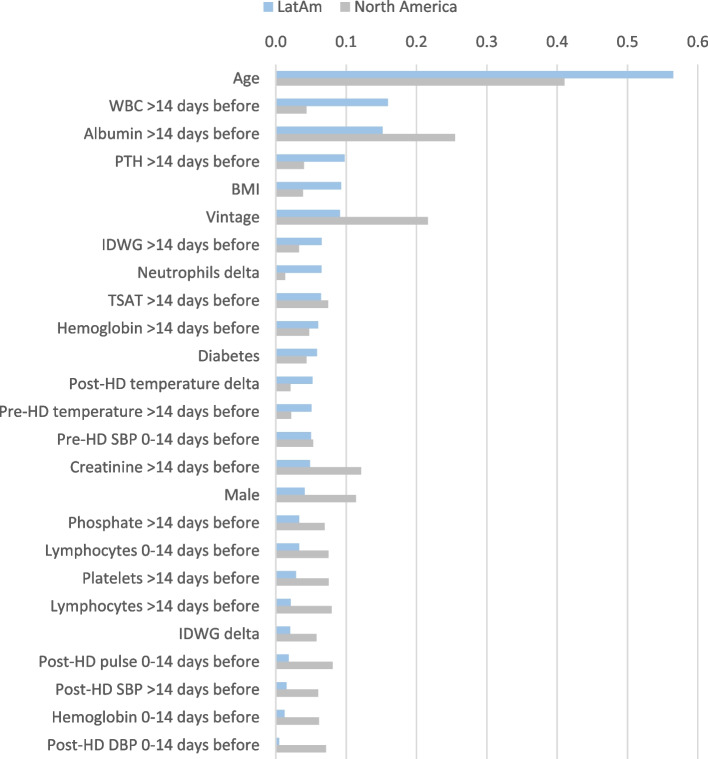
Regional importance of the top predictors of death any time after COVID-19 presentation. Mean SHAP values are shown for the top 15 predictors in each region, including the mutual and the exclusive top predictors in LatAm and North America. Mean SHAP values are show in descending order of importance in reference to the LatAm group

Albeit distinctions exist between world regions in the predictors of mortality any time after COVID-19 presentation, the trends in the top 15 predictors showed many consistent findings with older age, poorer nutrition (lower albumin and creatinine historically), longer vintage, lower TSAT levels historically, more inflammation (seen in LatAm by higher WBC counts historically and a change to a higher % of neutrophils and in North America by lower % of lymphocytes historically and at presentation) increasing the risk of death (Fig. 4). Some regional differences in the top predictors of mortality any time after COVID-19 included lower or missing iPTH historically and presence of diabetes being among the top 15 risk factors in only LatAm, while being male and higher post-HD pulse at presentation were only in the top 15 predictors in North America. Figure 5 shows a further regional comparison of the mean absolute SHAP values for the top 15 predictors of death any time after COVID-19 presentation from both regional cohorts, and Additional File 1; Supplementary Table 2 shows the SHAP values for all the predictors of death any time after COVID-19.

### Predictors of Shorter, Intermediate, and Longer-Term Death after COVID-19

**Fig. 6:**
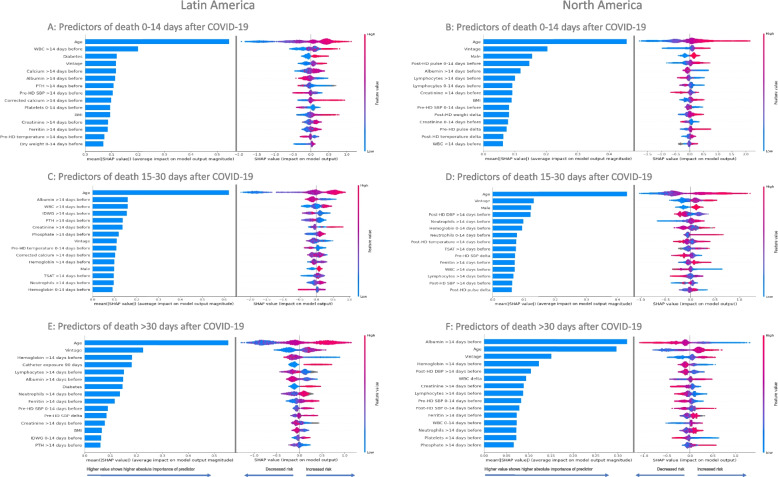
Top 15 predictors of death 0 to 14 days, 15 to 30 days, and > 30 days after COVID-19 presentation in descending order for the Latin America (**A**, **C**, **E**) and North America (**B**, **D**, **F**) cohorts. Bar plot in the left panels show the mean absolute SHAP values that estimate the average size of each variable’s contribution to predicting the outcome on the x-axis (calculated from the average absolute value for all patients). SHAP value plots in the right panels show the size and direction (more positive = higher risk or more negative = lower risk) of each variable’s influence on the outcome for each unique patient on the x-axis, with warmer colors representing higher observed values for that measurement, cooler colors indicating lower values for that measurement, and gray representing a missing value for that measurement. SHAP values are presented in the unit of log odds (i.e. logarithm of the odds ratio)

**Fig. 7:**
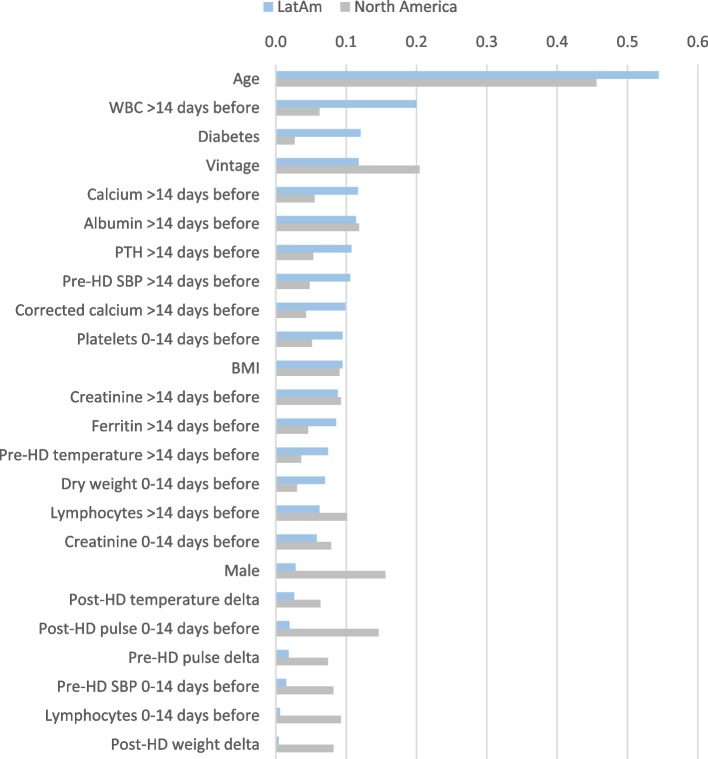
Regional importance of the predictors of death 0 to 14 days after COVID-19 presentation. Mean SHAP values are shown for the top 15 predictors in each region, including the mutual and the exclusive top predictors in LatAm and North America. Mean SHAP values are show in descending order of importance in reference to the LatAm group

Assessment of the top predictors of shorter-term death within specifically 0 to 14 days after COVID-19 presentation showed older age, higher WBC counts historically, longer vintage, lower albumin historically, higher BMI, and higher creatinine historically were among the top 15 risk factors for shorter-term mortality in both regions (Figs. 6 & 7, Additional File 1; Supplementary Table 3). Mineral bone disorder markers (lower or missing iPTH, higher calcium, higher corrected calcium) historically, higher ferritin levels historically, and having diabetes were found to only be in the top 15 predictors for short-term mortality in LatAm, while a higher post-HD pulse at presentation, a change to a higher pulse, and being male were only in the top 15 predictors in North America, among other distinctions.

**Fig. 8:**
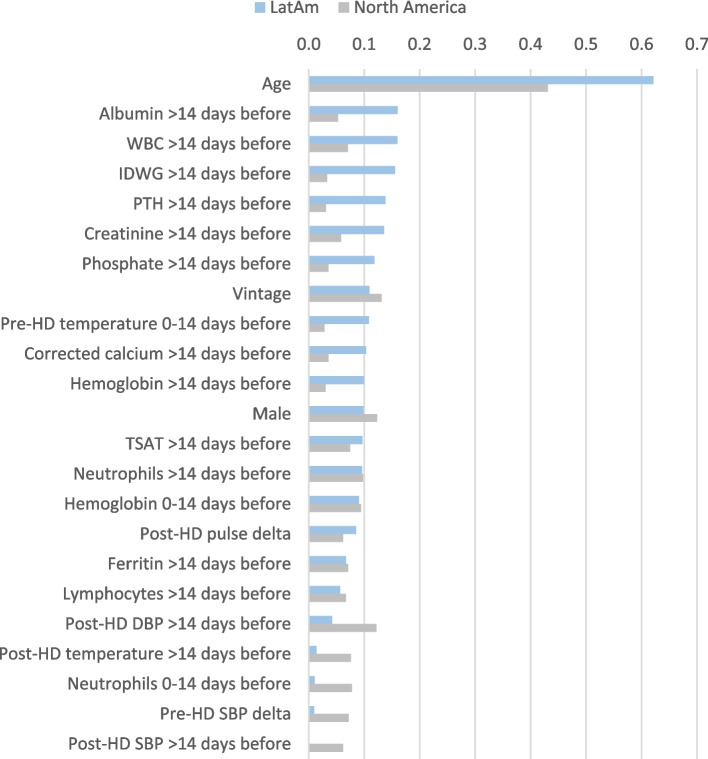
Regional importance of the predictors of death 15 to 30 days after COVID-19 presentation. Mean SHAP values are shown for the top 15 predictors in each region, including the mutual and the exclusive top predictors in LatAm and North America. Mean SHAP values are show in descending order of importance in reference to the LatAm group

The evaluation of the risk factors for intermediate-term mortality during 15 to 30 days after COVID-19 presentation identified consistencies in many of the top 15 predictors of death in between regions (older age, being male, higher TSAT and % of neutrophils historically, and hemoglobin at presentation), along with some regional heterogeneity in some factors (Figs. 6 & 8, Additional File 1; Supplementary Table 4). A surprising contrast in the predictors of mortality 15 to 30 days after COVID-19 between regions included higher WBC counts historically being a top predictor of death in LatAm, while this was opposite with lower WBC counts historically being a top predictor in North America. There was also an inverse association seen with shorter vintage being a top predictor of intermediate-term death in LatAm and vice versa in North America. BMI and diabetes were not among the top 15 predictors of intermediate-term mortality in either region.

**Fig. 9:**
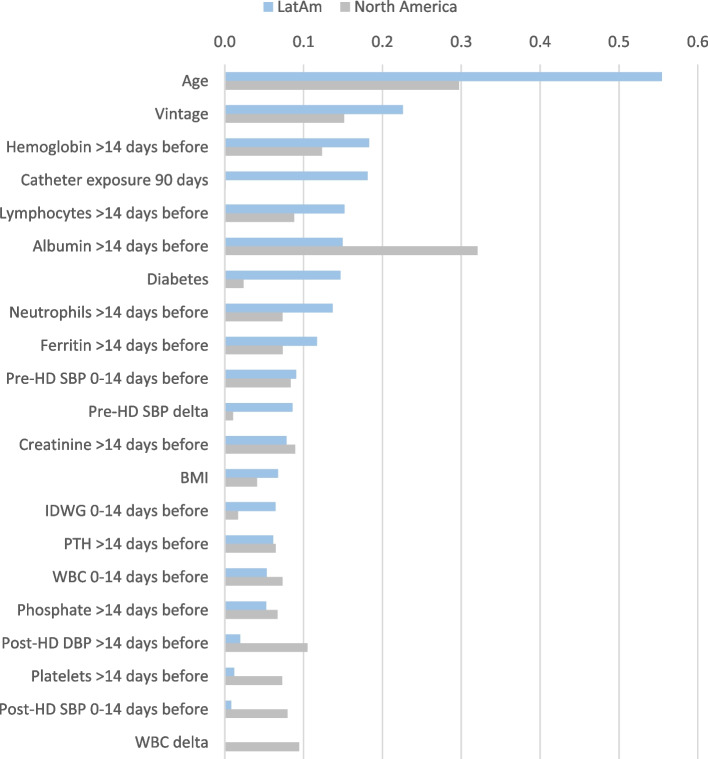
Regional importance of the predictors of death > 30 days after COVID-19 presentation. Mean SHAP values are shown for the top 15 predictors in each region, including the mutual and the exclusive top predictors in LatAm and North America. Mean SHAP values are show in descending order of importance in reference to the LatAm group

The examination of the predictors of longer-term mortality > 30 days after COVID-19 presentation found consistency in risk factors between regions for older age, longer dialysis vintage, lower hemoglobin levels historically, more inflammation (higher % of neutrophils and lower % of lymphocytes historically), poorer nutrition (lower albumin and creatinine historically), and higher ferritin levels being in the top 15 predictors (Fig. 6 & 9, Additional File 1; Supplementary Table 5). Interestingly, we found an inverse association between regions for pre-HD SBP at presentation with a higher SBP being a risk factor in LatAm and vice versa in North America. Catheter exposure for > 90 days, diabetes, lower PTH, and lower BMI were uniquely among the top 15 predictors of longer-term death in the LatAm cohort, as well as other factors. The demographic factor of sex was no longer among the top 15 predictors of a long-term death after COVID-19 in either region.

## Discussion

Among two regional cohorts of HD patients who presented with COVID-19 before SARS-CoV-2 vaccines were available, mortality any time after presentation was 8.3 percentage points higher in LatAm countries compared to the North American country of the United States. Shorter-term mortality after COVID-19 was more common in LatAm as compared to North America cohort, with the mortality rate being 7.7 and 3.3 percentage points higher within 14 days and during 15 to 30 days after presentation respectively. Conversely, longer-term mortality after COVID-19 was more frequent in North America, with the mortality rate being 2.7 percentage points higher than in the LatAm cohort. The series of machine learning models developed in parallel in each region were found to have suitable performance in prediction of death any time after COVID-19, as well as in the prespecified shorter-term follow up timeframes. Albeit we found suitable performance in the prediction of death events in prespecified intermediate- and longer-term periods in North America, the models did not perform as well in LatAm when considering AUPRC. This finding may be related to differences in the timing of outcomes and the number of patients used in the model development. We found some consistencies in top predictors of mortality after COVID-19 in LatAm and North America. In both regions, age and vintage were top predictors of death in all timeframes and the nutrition markers of albumin and creatinine were top predictors for every timeframe except 15–30 days after presentation. The top predictors of shorter-and intermediate-term mortality after COVID-19 appeared to include unique patient attributes (e.g. higher BMI and/or male sex) that were not top predictors for longer-term mortality. Despite the consistencies, there were several regional distinctions identified. Ultimately, the results showed patients who survived COVID-19 had a better clinical status historically and at presentation, which was clearly seen for markers of nutrition in all models at all follow up time points, and further included markers of anemia and mineral bone disorders. Achievement of quality targets before and throughout the recovery process may be of high importance to survival in COVID-19. Furthermore, markers of higher inflammation appeared to remarkably contribute to the risk of death and may be important to consider when determining a patient’s prognosis in COVID-19.

Our study is unique in that it used underexplored follow-up timeframes, included a wide variety of commonly reported variables in the world, assessed temporal patterns in clinical factors before COVID-19 presentation, and utilized machine learning techniques that can account for collinearity and missingness. Other efforts assessing the predictors of mortality in COVID-19 typically assessed outcomes about 30 to 90 days after presentation, and used traditional modeling techniques (e.g. regression methods) [25] that cannot handle a larger number of input variables and are prone to bias through confounding interactions [3, 4, 8]. These studies provided critical early insights to the nephrology community, yet further investigations with more follow up time and more generalizable patient numbers are sparse. In our study, we observed marked differences in most clinical and demographic factors between the groups who died or survived, which made the selection of meaningful predictors for traditional modeling efforts complex. Initial investigations of correlations and collinearity in our datasets found unacceptable interactions between most variables, and this led us to select machine learning techniques that can account for these issues and limit bias.

Previous studies investigating the risk factors for mortality in dialysis patients with COVID-19 have consistently found older age categories are one of the most important risk factors for death considering follow up timeframes of 28 to 90 days [3, 4, 8, 26]. Our findings in two regional cohorts of adult HD patients further substantiate these observations. In contrast with prior studies that commonly found presence of heart failure or ischemic heart disease to be a key predictor of mortality [3, 4, 27], we never found these to be in the top 15 predictors, in any model at any follow up period in either region. We presume this is reflective of the high importance of clinical variables (e.g. laboratories and vital signs) on the prediction of death after COVID-19, factors that were not included in other reports. The results of this study build upon insights from other studies in dialysis patients and ultimately provide unique results on clinical parameters, show important considerations in temporal associations, and used models that can avoid bias resulting from collinearity. Nonetheless, further analysis is needed to differentiate parameters that are attributable to risks in COVID-19, which would include comparing the predictors of mortality in patients with and without COVID-19.

Considering reports specifically from LatAm countries with longer follow up periods, a study of 741 HD patients with COVID-19 in Brazil showed 18.8% of patients died within 90 days of diagnosis in 2020, and the majority of death events were found to have occurred within 15 days [26]. Using a stepwise regression model, this study found the significant predictors of 90-day mortality in COVID-19 were diabetes and dialysis catheter use, in addition to increasing age in years [26]. We also observed diabetes was in the top 15 predictors of mortality any time, and during shorter- and longer-term follow up periods, after COVID-19 in LatAm. However, we only found catheter exposure was a risk factor for longer-term mortality, ultimately clarifying the that the risk factor is the most meaningful in the subset of patients who survive at least 30 days after COVID-19 in LatAm and may be specific to the region. Notably, we never found catheter exposure to be a top predictor of mortality after COVID-19 in the North America cohort. Given the Brazilian study only evaluated a limited number of predictors and did not include any laboratories or HD treatment variables, it may have inadvertently elevated associations with catheter use to appear more meaningful than they truly are considering the majority of the routinely captured clinical information [26].

Looking at reports specifically from North America with longer follow up periods, an analysis of data from 60,090 prevalent dialysis patients with COVID-19 in the United States who had Medicare insurance found 26.0% of patients died throughout 2020 [27]. This study used a Cox regression model to determine the risk factors related to mortality after COVID-19 diagnosis, and found the significant predictors of death included older age, longer dialysis vintage, being male, higher BMI categories, being of a white race, presence of congestive heart failure or ischemic heart disease along with other parameters (e.g. modality, population density, nursing home utilization). We showed consistent findings for increased risks of death in COVID-19 with older age and longer vintage for all follow up timepoints in our North America cohort. Further, we also found being male was a top predictor of mortality in COVID-19, especially for shorter- and intermediate-term outcomes. However, we did not observe male sex to be a top predictor of longer-term outcomes occurring > 30 days after presentation. We also found higher BMI to be a top predictor, yet only for shorter-term mortality within 14 days of presentation. Although BMI was not a top predictor of longer-term death in North America, it is noteworthy to mention that the association became inversed with lower BMI being associated with a higher risk of death coming in as the 34^th^ predictor in the region. Remarkably, this observation was more clearly seen in the LatAm cohort where higher BMI was among the top 15 predictors of shorter-term death and lower BMI was among the top 15 predictors of longer-term death after COVID-19 (Fig. 6). As mentioned earlier, we did not find heart failure or ischemic heart disease to be top predictors. We did not include race in our models since we focused on variables that are universally captured in both world regions; data on race is not captured in some LatAm countries, which is a limitation.

Traditional regression modelling techniques can provide a simpler interpretation on a population level due to the requirement for establishing a reference, with categories or successive changes in the measure, of which the former considers everyone in a group to be the same and the latter requires the assumption of linear relationships in effects [25]. This process allows a hazard ratio or odds ratio to be produced and provides an average probability of an outcome in one group or another, or by a specified increase/decrease. Although traditional techniques can provide a simple interpretation for a population, information gain is often lost, and unacceptable generalization can occur. Non-linear modeling, such as the machine learning techniques we utilized, can consider the effects for continuous variables without categorization and do not require arbitrary assumptions in linear relationships [25]. It is worthwhile to mention there have been advancements in predictive modeling techniques in recent years, and deep learning methods might have the potential to perform even better than the machine learning methods chosen by us due to the XGBoost model’s ability to account for collinearity and missingness [28, 29]. A limitation of these machine and deep learning models are that the outputs can be less intuitive on a population level. In our case, we report the SHAP values in log odds (i.e. the logarithm of the odds ratio) with average population risks being provided in absolute values that only show relative importance of a factor, yet not the direction of the association. Nonetheless, the individual predictions can provide more interpretable information for any given individual patient, in a more personalized manner, including each individual patient’s probability of experiencing an outcome, as well as the probability and direction of the association for each individual predictor variable for each individual patient. Importantly, the top predictors established consider the average risk for patients in each regional cohort and the top predictors for individuals will likely differ some since every affected patient may not have the same physiological disturbances in the same factors.

Although we observed consistencies in the top predictors of mortality in COVID-19 in HD patients between the world regions, we did find some contrasts in the top predictors as well as inverse associations. These could be in part reflective of the differences in the timing of death events after COVID, which occurred earlier in LatAm and later in North America. Supporting this, we did find some the top predictors of mortality changed from shorter to longer survival times, such as in the case of BMI. Also, these contrasts could be attributable to differences in the regional cohorts related to patient characteristics, practice patterns, and resource limitations. Some select laboratories were measured less frequently in Latin versus North America countries, which is a potential limitation. However, we did not qualitatively observe any concerning differences in the descriptive statistics for the cohorts.

Our findings highlight how machine learning techniques can provide personalized insights for individual patients to understand the specific risk factors of death in COVID-19 for each patient, as well as provide a better generalization of the most important risk factors for a cohort/population. We found most the models constructed had suitable performance in providing individualized prognosis for HD patients with COVID-19. These modeling techniques can be adopted by providers with analytical resources to assist care teams and enhance treatment paradigms. We recommend using an array of variables and including modifiable factors to provide potential ways to intervene. In the development of models, fewer variables could be considered, and data driven selection of variables is recommended. If models are adapted considering fewer variables (e.g. the top 15, 25, or 50), they would likely perform acceptably with the most information gain being attributable to the top predictors, yet a reasonable proportion of the top predictors should be included to maintain the ability to provide personalized predictions, especially for modifiable factors that can be intervened upon. Notably, we used a default cutoff threshold for calculation the balanced accuracy performance metric. It may be prudent to evaluate adjustments in this cutoff threshold for prospective efforts to optimize model performance for a specific use case and intervention.

Prior efforts have leveraged machine learning modeling to assist with early detection of SARS-CoV-2 infection in HD patients [30], and these models add another set of resources to be considered in the clinician’s toolbox by providing a method to suitably assist with the prognosis of HD patients who contract COVID-19. Amidst the time of SARS-CoV-2 vaccines being more and more of an option in the world, the predictors of mortality will need to be established specifically in vaccinated dialysis patients considering regional differences in the world in patient populations and vaccine types. Given some countries continue to have limitations in access to SARS-CoV-2 vaccines [12], these models and the established predictors of mortality in HD patients before vaccines were available will be of high importance to the global nephrology community and can be leveraged for the development of models in vaccinated cohorts.

## Conclusions

In summary, our findings show the profiles of mortality in HD patients with COVID-19 were distinct in LatAm and North America throughout the year 2020. There was a higher mortality rate within 0–14 or 15–30 days after COVID-19 in LatAm, while the mortality rate was higher in North America > 30 days after presentation. Irrespective of these differences, a marked proportion of HD patients died > 30 days after presentation with COVID-19 (6% in LatAm and 9% in North America cohorts). We were able to successfully construct a series of prediction models with suitable performance in both regions for determining the risk of death in an HD patient any time after COVID-19 presentation, as well as within 0–14, 15–30, and > 30 days after COVID-19 presentation. Results showed older age, longer vintage, poor nutrition, and higher inflammation were consistently top predictors of death in COVID-19 in both world regions at all timepoints after COVID-19 presentation. Unique patient attributes including higher BMI and male sex were top predictors of shorter-and intermediate-term mortality, yet not longer-term mortality. These insights further expand our understanding of the profiles and predictors of mortality and provide modeling techniques that can be considered for use by dialysis providers internationally.

## Supplementary Information

**Additional file 1: Supplementary Table 1.** Initial, Tuning Range, and Final Hyperparameter Settings for Models. **Supplementary Table 2.** Mean SHAP values for all predictors of death any time after COVID-19 presentation, by region. **Supplementary Table 3.** Mean SHAP values for all predictors of death 0-14 days after COVID-19 presentation, by region. **Supplementary Table 4.** Mean SHAP values for all predictors of death 15-30 days after COVID-19 presentation, by region. **Supplementary Table 5.** Mean SHAP values for all predictors of death >30 days after COVID-19 presentation, by region.

## Data Availability

The datasets generated and/or analysed during the current study are not publicly available due to the datasets being captured from private electronic medical record systems that are restricted to use by only authorized employees of Fresenius Medical Care, but are available from the corresponding author on reasonable request. A reasonable request to access the datasets would include and require agreements to be established between Fresenius Medical Care and an external individual(s) institution.

## Abbreviations

AUC: Area under the curve
BMI: Body mass index
COVID-19: Coronavirus disease
DBP: Diastolic blood pressure
HD: Hemodialysis
IDWG: Interdialytic weight gain
LatAm: Latin America
iPTH: Intact parathyroid hormone
SARS-CoV-2: Severe Acute Respiratory Syndrome Coronavirus 2
SBP: Systolic blood pressure
SD: Standard deviation
SHAP: Shapley
TSAT: Transferrin saturation
WBC: White blood cell
