# Supplementary material for: Predictors of shorter- and longer-term mortality after COVID-19 presentation among dialysis patients: parallel use of machine learning models in Latin and North American countries
Source: BMC Nephrol. 2022 Oct 22;23:340. doi: 10.1186/s12882-022-02961-x (PMC9587666; doi:10.1186/s12882-022-02961-x)
Supplement: Supplementary file 1 — Additional file 1: Supplementary Table 1. Initial, Tuning Range, and Final Hyperparameter Settings for Models. Supplementary Table 2. Mean SHAP values for all predictors of death any time after COVID-19 presentation, by region. Supplementary Table 3. Mean SHAP values for all predictors of death 0-14 days after COVID-19 presentation, by region. Supplementary Table 4. Mean SHAP values for all predictors of death 15-30 days after COVID-19 presentation, by region. Supplementary Table 5. Mean SHAP values for all predictors of death >30 days after COVID-19 presentation, by region. [file 12882_2022_2961_MOESM1_ESM.docx]

**Additional File 1: Supplementary Materials:**

| Supplementary Table 1: Initial, Tuning Range, and Final Hyperparameter Settings for Models | | | |
| --- | --- | --- | --- |
| Model Parameter | **Initial** | **Tuning Range** | **Final*** |
| Number of trees | 1000 | 1000 | 1000 |
| Learning rate | 0.1 | 0.1 | 0.1 |
| Maximum depth per tree | 3 | 3,4,5,6,7 | 4 |
| Subsample of columns per tree | 0.5 | 0.5, 0.7, 0.8, 0.9 | 0.8 |
| Subsample per tree | 0.5 | 0.5, 0.7, 0.8, 0.9 | 0.8 |
| Minimum child weight | 5 | 1,2,5,10,20,50 | 5 |
| Alpha | 5 | 1,3,5 | 5 |
| Gamma | 5 | 1,3,5 | 5 |
| Lambda | 5 | 1,3,5 | 5 |
| Objective | Binary logistic | Binary logistic | Binary logistic |
| Evaluation metric | AUPRC, log loss | AUPRC, log loss | AUPRC, log loss |
| Missing | Not a number | Not a number | Not a number |
| Number of boost rounds | 500 | 500 | 500 |
| The same initial model parameters and tuning ranges were considered in both the Latin America (LatAm) and North America cohorts. Hyperparameter tuning was performed for the models that predicted mortality any time after COVID-19 in the overall cohort in each region.  *The models that predicted mortality any time after COVID-19 produced the same final hyperparameter settings for the LatAm and North America cohorts. These hyperparameter settings were universally applied to the sub-analysis models that predicted mortality within 0-14 days, 15-30 days, and >30 days after COVID-19. | | | |

| Supplementary Table 2: Mean SHAP values for all predictors of death any time after COVID-19 presentation, by region | | |
| --- | --- | --- |
| **Predictors** | **LatAm** | **North America** |
| Age | 0.564965 | 0.410258 |
| WBC >14 days before | 0.159414 | 0.043457 |
| Albumin >14 days before | 0.151595 | 0.254868 |
| PTH >14 days before | 0.097715 | 0.039768 |
| BMI | 0.092997 | 0.038420 |
| Vintage | 0.090945 | 0.216013 |
| IDWG >14 days before | 0.065147 | 0.032632 |
| Neutrophils delta | 0.064696 | 0.013206 |
| TSAT >14 days before | 0.063951 | 0.074393 |
| Hemoglobin >14 days before | 0.060160 | 0.047428 |
| Diabetes | 0.058206 | 0.043277 |
| Post-HD temperature delta | 0.051926 | 0.020820 |
| Pre-HD temperature >14 days before | 0.050547 | 0.021751 |
| Pre-HD SBP 0-14 days before | 0.049786 | 0.052942 |
| Creatinine >14 days before | 0.048524 | 0.121349 |
| Calcium >14 days before | 0.048267 | 0.031083 |
| Ferritin >14 days before | 0.048039 | 0.051632 |
| Calcium delta | 0.043491 | 0.024634 |
| Pre-HD weight delta | 0.042515 | 0.021042 |
| Corrected calcium >14 days before | 0.041821 | 0.032150 |
| Male | 0.041091 | 0.113758 |
| Post-HD pulse >14 days before | 0.040111 | 0.037232 |
| Pre-HD pulse 0-14 days before | 0.039964 | 0.038629 |
| Calcium 0-14 days before | 0.037029 | 0.017937 |
| Post-HD DBP delta | 0.035777 | 0.025052 |
| Neutrophils >14 days before | 0.035267 | 0.032866 |
| Phosphate >14 days before | 0.033072 | 0.069435 |
| Lymphocytes 0-14 days before | 0.032948 | 0.074467 |
| Catheter exposure 180 days | 0.031274 | 0.003554 |
| Pre-HD temperature 0-14 days before | 0.029940 | 0.044323 |
| Platelets >14 days before | 0.028322 | 0.075128 |
| Pre-HD DBP delta | 0.028172 | 0.028539 |
| WBC 0-14 days before | 0.027948 | 0.029130 |
| Platelets 0-14 days before | 0.026691 | 0.021896 |
| Pre-HD pulse delta | 0.026157 | 0.048014 |
| Pre-HD SBP delta | 0.025490 | 0.020544 |
| TSAT delta | 0.024759 | 0.019914 |
| Albumin delta | 0.024583 | 0.006383 |
| WBC delta | 0.024155 | 0.049683 |
| Corrected calcium delta | 0.023807 | 0.010766 |
| Pre-HD temperature delta | 0.022160 | 0.045502 |
| Post-HD weight >14 days before | 0.021979 | 0.013972 |
| Hypertension | 0.021421 | 0.012894 |
| Lymphocytes >14 days before | 0.020941 | 0.079270 |
| IDWG delta | 0.020286 | 0.057559 |
| Pre-HD weight >14 days before | 0.019870 | 0.018956 |
| Phosphate 0-14 days before | 0.019084 | 0.024807 |
| PTH 0-14 days before | 0.019058 | 0.015326 |
| Post-HD pulse 0-14 days before | 0.018032 | 0.080555 |
| Albumin 0-14 days before | 0.017833 | 0.030483 |
| Catheter exposure 120 days | 0.017561 | 0.001381 |
| Post-HD weight 0-14 days before | 0.016818 | 0.014879 |
| Catheter exposure 90 days | 0.016278 | 0.001565 |
| Pre-HD DBP 0-14 days before | 0.015411 | 0.039837 |
| Post-HD SBP >14 days before | 0.014902 | 0.060281 |
| Post-HD pulse delta | 0.014604 | 0.027136 |
| Pre-HD pulse >14 days before | 0.013862 | 0.034696 |
| Post-HD DBP >14 days before | 0.013125 | 0.021231 |
| Pre-HD SBP >14 days before | 0.012609 | 0.037338 |
| Post-HD weight delta | 0.012389 | 0.046775 |
| Hemoglobin 0-14 days before | 0.012132 | 0.061009 |
| Dry weight >14 days before | 0.011937 | 0.008558 |
| Dry weight 0-14 days before | 0.011726 | 0.028988 |
| PTH delta | 0.010928 | 0.020325 |
| TSAT 0-14 days before | 0.010279 | 0.025264 |
| Creatinine 0-14 days before | 0.010252 | 0.025917 |
| Post-HD temperature >14 days before | 0.010024 | 0.033169 |
| Post-HD SBP 0-14 days before | 0.009981 | 0.030796 |
| Platelets delta | 0.008970 | 0.021022 |
| IDWG 0-14 days before | 0.008195 | 0.015504 |
| Creatinine delta | 0.008036 | 0.034981 |
| Phosphate delta | 0.007882 | 0.021884 |
| Lymphocytes delta | 0.007745 | 0.018193 |
| Post-HD SBP delta | 0.007171 | 0.035145 |
| Hemoglobin delta | 0.006341 | 0.028174 |
| Neutrophils 0-14 days before | 0.006054 | 0.026444 |
| Ferritin delta | 0.005899 | 0.015710 |
| Pre-HD DBP >14 days before | 0.005895 | 0.035623 |
| Ferritin 0-14 days before | 0.005718 | 0.014607 |
| Pre-HD weight 0-14 days before | 0.005489 | 0.015509 |
| Post-HD DBP 0-14 days before | 0.004850 | 0.070963 |
| Dry weight delta | 0.003502 | 0.019116 |
| Corrected calcium 0-14 days before | 0.003266 | 0.015938 |
| Post-HD temperature 0-14 days before | 0.001387 | 0.033076 |
| Heart failure | 0.000000 | 0.039220 |
| COPD | 0.000000 | 0.009905 |
| Ischemic heart disease | 0.000000 | 0.009015 |
| Cancer | 0.000000 | 0.001225 |
| Liver disease | 0.000000 | 0.001133 |
| Mean SHAP values are show in descending order of importance in reference to the Latin America group. | | |

| Supplementary Table 3: Mean SHAP values for all predictors of death 0-14 days after COVID-19 presentation, by region | | |
| --- | --- | --- |
| **Predictors** | **LatAm** | **North America** |
| Age | 0.544373 | 0.456058 |
| WBC >14 days before | 0.199663 | 0.061876 |
| Diabetes | 0.120387 | 0.026674 |
| Vintage | 0.117516 | 0.204220 |
| Calcium >14 days before | 0.116590 | 0.054968 |
| Albumin >14 days before | 0.113766 | 0.118393 |
| PTH >14 days before | 0.107509 | 0.053193 |
| Pre-HD SBP >14 days before | 0.105740 | 0.047898 |
| Corrected calcium >14 days before | 0.099016 | 0.042749 |
| Platelets 0-14 days before | 0.095007 | 0.051209 |
| BMI | 0.094718 | 0.090460 |
| Creatinine >14 days before | 0.087803 | 0.092419 |
| Ferritin >14 days before | 0.085579 | 0.045798 |
| Pre-HD temperature >14 days before | 0.073973 | 0.035986 |
| Dry weight 0-14 days before | 0.069603 | 0.029884 |
| WBC delta | 0.062964 | 0.041957 |
| Lymphocytes >14 days before | 0.061739 | 0.101400 |
| Phosphate >14 days before | 0.060321 | 0.043125 |
| Post-HD pulse >14 days before | 0.059258 | 0.055261 |
| Creatinine 0-14 days before | 0.058172 | 0.078497 |
| Platelets >14 days before | 0.055658 | 0.061565 |
| Phosphate 0-14 days before | 0.054013 | 0.035485 |
| Neutrophils >14 days before | 0.049306 | 0.035446 |
| Neutrophils delta | 0.048788 | 0.016789 |
| Pre-HD temperature 0-14 days before | 0.048532 | 0.020517 |
| IDWG >14 days before | 0.047054 | 0.061153 |
| Hemoglobin >14 days before | 0.046408 | 0.041710 |
| TSAT >14 days before | 0.045564 | 0.028050 |
| Calcium delta | 0.038456 | 0.019886 |
| Dry weight >14 days before | 0.037723 | 0.020513 |
| Post-HD SBP 0-14 days before | 0.037721 | 0.060878 |
| Pre-HD weight delta | 0.036115 | 0.035916 |
| Post-HD DBP delta | 0.032296 | 0.042174 |
| IDWG 0-14 days before | 0.031465 | 0.059391 |
| Hypertension | 0.029898 | 0.000000 |
| Pre-HD weight >14 days before | 0.029730 | 0.035640 |
| Pre-HD DBP delta | 0.027855 | 0.058180 |
| Male | 0.027773 | 0.155631 |
| Hemoglobin 0-14 days before | 0.027597 | 0.053113 |
| Corrected calcium delta | 0.026470 | 0.001187 |
| Post-HD temperature delta | 0.026093 | 0.063371 |
| WBC 0-14 days before | 0.025964 | 0.020004 |
| Post-HD weight 0-14 days before | 0.024617 | 0.015821 |
| Pre-HD pulse >14 days before | 0.023943 | 0.039147 |
| TSAT delta | 0.022890 | 0.030063 |
| Platelets delta | 0.021716 | 0.051441 |
| Pre-HD pulse 0-14 days before | 0.020559 | 0.054487 |
| Neutrophils 0-14 days before | 0.019754 | 0.049316 |
| Post-HD DBP 0-14 days before | 0.019656 | 0.030679 |
| Creatinine delta | 0.019378 | 0.032195 |
| Pre-HD weight 0-14 days before | 0.019365 | 0.018487 |
| Pre-HD SBP delta | 0.019285 | 0.030621 |
| Post-HD pulse 0-14 days before | 0.019281 | 0.146227 |
| Post-HD DBP >14 days before | 0.018230 | 0.044909 |
| Post-HD pulse delta | 0.018098 | 0.051079 |
| Pre-HD pulse delta | 0.017999 | 0.074182 |
| Calcium 0-14 days before | 0.017156 | 0.022597 |
| Post-HD weight >14 days before | 0.016305 | 0.011286 |
| Pre-HD DBP >14 days before | 0.016177 | 0.026303 |
| Hemoglobin delta | 0.015944 | 0.036235 |
| IDWG delta | 0.015783 | 0.049949 |
| Pre-HD SBP 0-14 days before | 0.014754 | 0.081961 |
| Albumin 0-14 days before | 0.013582 | 0.061574 |
| Heart failure | 0.013461 | 0.040646 |
| Albumin delta | 0.012793 | 0.024842 |
| PTH 0-14 days before | 0.012264 | 0.029139 |
| Pre-HD temperature delta | 0.012257 | 0.044573 |
| Phosphate delta | 0.012070 | 0.035858 |
| Pre-HD DBP 0-14 days before | 0.011997 | 0.039820 |
| Post-HD SBP delta | 0.011319 | 0.029397 |
| Corrected calcium 0-14 days before | 0.011150 | 0.016048 |
| Catheter exposure 180 days | 0.009773 | 0.006563 |
| Catheter exposure 120 days | 0.008867 | 0.004513 |
| Lymphocytes 0-14 days before | 0.005779 | 0.092491 |
| Post-HD temperature 0-14 days before | 0.005756 | 0.048520 |
| Post-HD SBP >14 days before | 0.004986 | 0.046278 |
| Post-HD weight delta | 0.004037 | 0.081592 |
| TSAT 0-14 days before | 0.004021 | 0.010843 |
| Post-HD temperature >14 days before | 0.000000 | 0.046624 |
| Lymphocytes delta | 0.000000 | 0.044415 |
| PTH delta | 0.000000 | 0.037210 |
| Ferritin 0-14 days before | 0.000000 | 0.032414 |
| Ferritin delta | 0.000000 | 0.021338 |
| Dry weight delta | 0.000000 | 0.006599 |
| COPD | 0.000000 | 0.002721 |
| Catheter exposure 90 days | 0.000000 | 0.002079 |
| Ischemic heart disease | 0.000000 | 0.000000 |
| Cancer | 0.000000 | 0.000000 |
| Liver disease | 0.000000 | 0.000000 |
| Mean SHAP values are show in descending order of importance in reference to the Latin America group. | | |

| Supplementary Table 4: Mean SHAP values for all predictors of death 15-30 days after COVID-19 presentation, by region | | |
| --- | --- | --- |
| **Predictors** | **LatAm** | **North America** |
| Age | 0.621402 | 0.431044 |
| Albumin >14 days before | 0.160314 | 0.053000 |
| WBC >14 days before | 0.159887 | 0.070682 |
| IDWG >14 days before | 0.155830 | 0.032969 |
| PTH >14 days before | 0.138217 | 0.030697 |
| Creatinine >14 days before | 0.135703 | 0.058239 |
| Phosphate >14 days before | 0.118346 | 0.035389 |
| Vintage | 0.109407 | 0.131401 |
| Pre-HD temperature 0-14 days before | 0.108307 | 0.028273 |
| Corrected calcium >14 days before | 0.103366 | 0.035093 |
| Hemoglobin >14 days before | 0.099379 | 0.030603 |
| Male | 0.098716 | 0.123440 |
| TSAT >14 days before | 0.096640 | 0.074611 |
| Neutrophils >14 days before | 0.096190 | 0.097913 |
| Hemoglobin 0-14 days before | 0.090399 | 0.094326 |
| Post-HD pulse delta | 0.085554 | 0.062070 |
| Calcium >14 days before | 0.079326 | 0.021049 |
| BMI | 0.068779 | 0.037069 |
| Ferritin >14 days before | 0.066698 | 0.070799 |
| Platelets delta | 0.057063 | 0.011296 |
| Lymphocytes >14 days before | 0.056515 | 0.066701 |
| Pre-HD SBP 0-14 days before | 0.053784 | 0.014327 |
| Pre-HD DBP delta | 0.052504 | 0.020507 |
| Post-HD weight >14 days before | 0.047156 | 0.018238 |
| Hypertension | 0.046567 | 0.032464 |
| Dry weight 0-14 days before | 0.043616 | 0.009349 |
| Post-HD DBP >14 days before | 0.042171 | 0.121902 |
| Pre-HD DBP 0-14 days before | 0.038626 | 0.026921 |
| Hemoglobin delta | 0.036781 | 0.039122 |
| Pre-HD pulse delta | 0.036404 | 0.039505 |
| Pre-HD temperature >14 days before | 0.036186 | 0.013363 |
| Phosphate 0-14 days before | 0.035438 | 0.015634 |
| Calcium 0-14 days before | 0.034785 | 0.006128 |
| Pre-HD weight delta | 0.034430 | 0.040106 |
| Calcium delta | 0.033451 | 0.008810 |
| PTH delta | 0.033105 | 0.050599 |
| Pre-HD temperature delta | 0.031944 | 0.027061 |
| Pre-HD pulse >14 days before | 0.030921 | 0.010915 |
| Phosphate delta | 0.029722 | 0.019106 |
| Dry weight >14 days before | 0.028889 | 0.016787 |
| Lymphocytes 0-14 days before | 0.026702 | 0.039855 |
| IDWG 0-14 days before | 0.026462 | 0.053572 |
| Post-HD temperature delta | 0.025880 | 0.023876 |
| Pre-HD weight 0-14 days before | 0.023780 | 0.021454 |
| Post-HD temperature 0-14 days before | 0.023535 | 0.040143 |
| Corrected calcium delta | 0.023482 | 0.021926 |
| Post-HD DBP delta | 0.023257 | 0.038610 |
| WBC 0-14 days before | 0.023220 | 0.016194 |
| Post-HD pulse >14 days before | 0.022602 | 0.037663 |
| Pre-HD weight >14 days before | 0.021437 | 0.031029 |
| PTH 0-14 days before | 0.019875 | 0.010170 |
| Pre-HD DBP >14 days before | 0.019751 | 0.032469 |
| Platelets >14 days before | 0.019540 | 0.046196 |
| WBC delta | 0.018196 | 0.041859 |
| Platelets 0-14 days before | 0.016889 | 0.006848 |
| Creatinine 0-14 days before | 0.015057 | 0.040552 |
| Pre-HD pulse 0-14 days before | 0.014216 | 0.036027 |
| Post-HD temperature >14 days before | 0.013628 | 0.076048 |
| Pre-HD SBP >14 days before | 0.013100 | 0.049744 |
| Post-HD SBP delta | 0.011556 | 0.060486 |
| Post-HD weight 0-14 days before | 0.011016 | 0.021080 |
| Neutrophils 0-14 days before | 0.010468 | 0.077909 |
| Pre-HD SBP delta | 0.009968 | 0.071725 |
| Post-HD weight delta | 0.009912 | 0.034877 |
| Post-HD SBP 0-14 days before | 0.009332 | 0.020123 |
| Lymphocytes delta | 0.008911 | 0.010477 |
| Catheter exposure 90 days | 0.008350 | 0.000000 |
| Post-HD DBP 0-14 days before | 0.007749 | 0.044647 |
| Neutrophils delta | 0.006694 | 0.020528 |
| Post-HD SBP >14 days before | 0.000000 | 0.062210 |
| Post-HD pulse 0-14 days before | 0.000000 | 0.056406 |
| Ferritin 0-14 days before | 0.000000 | 0.045969 |
| Dry weight delta | 0.000000 | 0.033409 |
| Albumin 0-14 days before | 0.000000 | 0.026963 |
| TSAT 0-14 days before | 0.000000 | 0.026274 |
| IDWG delta | 0.000000 | 0.024183 |
| Ferritin delta | 0.000000 | 0.022996 |
| Corrected calcium 0-14 days before | 0.000000 | 0.021964 |
| TSAT delta | 0.000000 | 0.017890 |
| Creatinine delta | 0.000000 | 0.011435 |
| Liver disease | 0.000000 | 0.007144 |
| Heart failure | 0.000000 | 0.006163 |
| Catheter exposure 180 days | 0.000000 | 0.004738 |
| Diabetes | 0.000000 | 0.004401 |
| Ischemic heart disease | 0.000000 | 0.003854 |
| Albumin delta | 0.000000 | 0.002648 |
| Cancer | 0.000000 | 0.000000 |
| COPD | 0.000000 | 0.000000 |
| Catheter exposure 120 days | 0.000000 | 0.000000 |
| Mean SHAP values are show in descending order of importance in reference to the Latin America group. | | |

| Supplementary Table 5: Mean SHAP values for all predictors of death >30 days after COVID-19 presentation, by region | | |
| --- | --- | --- |
| **Predictors** | **LatAm** | **North America** |
| Age | 0.554469 | 0.297155 |
| Vintage | 0.225846 | 0.151323 |
| Hemoglobin >14 days before | 0.182973 | 0.123221 |
| Catheter exposure 90 days | 0.181110 | 0.001316 |
| Lymphocytes >14 days before | 0.151855 | 0.088000 |
| Albumin >14 days before | 0.149412 | 0.320681 |
| Diabetes | 0.146826 | 0.023627 |
| Neutrophils >14 days before | 0.136660 | 0.073015 |
| Ferritin >14 days before | 0.116915 | 0.073427 |
| Pre-HD SBP 0-14 days before | 0.090461 | 0.083482 |
| Pre-HD SBP delta | 0.085714 | 0.010394 |
| Creatinine >14 days before | 0.078297 | 0.089120 |
| BMI | 0.067424 | 0.040986 |
| IDWG 0-14 days before | 0.064078 | 0.016819 |
| PTH >14 days before | 0.061416 | 0.064737 |
| Phosphate 0-14 days before | 0.056013 | 0.031046 |
| WBC 0-14 days before | 0.053148 | 0.073113 |
| Phosphate >14 days before | 0.052612 | 0.066813 |
| Post-HD DBP 0-14 days before | 0.050600 | 0.028461 |
| Post-HD weight >14 days before | 0.048339 | 0.027022 |
| Pre-HD weight >14 days before | 0.043480 | 0.024745 |
| Pre-HD weight 0-14 days before | 0.042683 | 0.021399 |
| TSAT >14 days before | 0.039120 | 0.054412 |
| Calcium delta | 0.038724 | 0.028809 |
| Pre-HD DBP >14 days before | 0.038100 | 0.051350 |
| Post-HD SBP delta | 0.038090 | 0.050825 |
| Pre-HD weight delta | 0.036505 | 0.030673 |
| WBC >14 days before | 0.035212 | 0.054924 |
| Pre-HD temperature 0-14 days before | 0.035040 | 0.043775 |
| Corrected calcium 0-14 days before | 0.034351 | 0.016137 |
| Neutrophils 0-14 days before | 0.032599 | 0.032555 |
| IDWG delta | 0.030234 | 0.060081 |
| IDWG >14 days before | 0.028772 | 0.034976 |
| Post-HD pulse delta | 0.028733 | 0.013100 |
| Corrected calcium >14 days before | 0.028112 | 0.046517 |
| Post-HD weight 0-14 days before | 0.025400 | 0.020411 |
| Dry weight 0-14 days before | 0.024856 | 0.015833 |
| Pre-HD temperature >14 days before | 0.023904 | 0.019773 |
| Pre-HD pulse 0-14 days before | 0.023858 | 0.062374 |
| Platelets 0-14 days before | 0.023382 | 0.034858 |
| Pre-HD DBP delta | 0.020266 | 0.018106 |
| Albumin 0-14 days before | 0.020020 | 0.043371 |
| Pre-HD SBP >14 days before | 0.019853 | 0.030189 |
| Hemoglobin 0-14 days before | 0.019715 | 0.040885 |
| Post-HD DBP >14 days before | 0.019683 | 0.104879 |
| Pre-HD pulse delta | 0.019392 | 0.029133 |
| Calcium >14 days before | 0.018626 | 0.013498 |
| Male | 0.016876 | 0.001111 |
| Post-HD temperature >14 days before | 0.016142 | 0.025962 |
| Post-HD pulse 0-14 days before | 0.015529 | 0.031355 |
| Pre-HD temperature delta | 0.015326 | 0.021346 |
| Post-HD weight delta | 0.014768 | 0.029308 |
| Platelets delta | 0.014575 | 0.025918 |
| Post-HD DBP delta | 0.013326 | 0.018801 |
| Platelets >14 days before | 0.011982 | 0.072873 |
| Hemoglobin delta | 0.011857 | 0.046390 |
| Post-HD temperature 0-14 days before | 0.009365 | 0.018213 |
| Post-HD SBP 0-14 days before | 0.008160 | 0.079418 |
| Hypertension | 0.008142 | 0.005264 |
| Phosphate delta | 0.007975 | 0.019544 |
| Calcium 0-14 days before | 0.007374 | 0.011042 |
| Post-HD pulse >14 days before | 0.006251 | 0.036120 |
| Pre-HD DBP 0-14 days before | 0.005164 | 0.041712 |
| Creatinine 0-14 days before | 0.000000 | 0.057301 |
| Pre-HD pulse >14 days before | 0.000000 | 0.041266 |
| Ischemic heart disease | 0.000000 | 0.041254 |
| PTH 0-14 days before | 0.000000 | 0.034692 |
| Creatinine delta | 0.000000 | 0.030829 |
| Dry weight delta | 0.000000 | 0.029402 |
| Dry weight >14 days before | 0.000000 | 0.028977 |
| Neutrophils delta | 0.000000 | 0.027652 |
| Lymphocytes delta | 0.000000 | 0.023175 |
| Ferritin delta | 0.000000 | 0.017748 |
| Post-HD SBP >14 days before | 0.000000 | 0.017037 |
| Post-HD temperature delta | 0.000000 | 0.016752 |
| PTH delta | 0.000000 | 0.016378 |
| TSAT 0-14 days before | 0.000000 | 0.016059 |
| Corrected calcium delta | 0.000000 | 0.015621 |
| Albumin delta | 0.000000 | 0.013847 |
| Heart failure | 0.000000 | 0.012478 |
| Lymphocytes 0-14 days before | 0.000000 | 0.011005 |
| Ferritin 0-14 days before | 0.000000 | 0.010103 |
| TSAT delta | 0.000000 | 0.007274 |
| Liver disease | 0.000000 | 0.007001 |
| COPD | 0.000000 | 0.002611 |
| WBC delta | 0.000000 | 0.094200 |
| Cancer | 0.000000 | 0.000000 |
| Catheter exposure 120 days | 0.000000 | 0.000000 |
| Catheter exposure 180 days | 0.000000 | 0.000000 |
| Mean SHAP values are show in descending order of importance in reference to the Latin America group. | | |
